# Seasonal divergence in the interannual responses of Northern Hemisphere vegetation activity to variations in diurnal climate

**DOI:** 10.1038/srep19000

**Published:** 2016-01-11

**Authors:** Xiuchen Wu, Hongyan Liu, Xiaoyan Li, Eryuan Liang, Pieter S. A. Beck, Yongmei Huang

**Affiliations:** 1 State Key Laboratory of Earth Processes and Resource Ecology, Beijing Normal University, Beijing, 100875, China; 2 Joint Center for Global Change Studies (JCGCS), Beijing, 100875, China; 3 College of Resources Science and Technology, Beijing Normal University, Beijing, 100875, China; 4 College of Urban and Environmental Science, Peking University, Beijing, 100871, China; 5 Key Laboratory of Tibetan Environment Changes and Land Surface Processes, Institute of Tibetan Plateau Research, Chinese Academy of Sciences, Beijing, 100085, China; 6 Forest Resources and Climate Unit, Institute for Environment and Sustainability (IES), Joint Research Centre (JRC), European Commission, Ispra, VA, Italy

## Abstract

Seasonal asymmetry in the interannual variations in the daytime and nighttime climate in the Northern Hemisphere (NH) is well documented, but its consequences for vegetation activity remain poorly understood. Here, we investigate the interannual responses of vegetation activity to variations of seasonal mean daytime and nighttime climate in NH (>30 °N) during the past decades using remote sensing retrievals, FLUXNET and tree ring data. Despite a generally significant and positive response of vegetation activity to seasonal mean maximum temperature (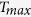) in ~22–25% of the boreal (>50 °N) NH between spring and autumn, spring-summer progressive water limitations appear to decouple vegetation activity from the mean summer 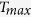, particularly in climate zones with dry summers. Drought alleviation during autumn results in vegetation recovery from the marked warming-induced drought limitations observed in spring and summer across 24–26% of the temperate NH. Vegetation activity exhibits a pervasively negative correlation with the autumn mean minimum temperature, which is in contrast to the ambiguous patterns observed in spring and summer. Our findings provide new insights into how seasonal asymmetry in the interannual variations in the mean daytime and nighttime climate interacts with water limitations to produce spatiotemporally variable responses of vegetation growth.

The vegetation activity in temperate and cold regions is strongly controlled by climate, which is causing rapid changes in the vegetation activity in the Northern Hemisphere (NH) as the Earth warms^1^,^2^,^3^,^4^. Satellite observations, Earth system models and atmospheric inversions suggest that growing-season warming has increased the photosynthetic activity of the NH terrestrial vegetation during past decades^1^,^2^,^4^ to a greater extent than other environmental processes (e.g., CO_2_ and nitrogen fertilization)^5^. However, the responses of vegetation to climate warming are not uniform in space or time; covariation between the growing season temperature and vegetation activity varies regionally within the NH and appears to be weakening in many regions^6^. Simultaneously, mean daytime and nighttime warming during the growing-season produce different responses in vegetation productivity in the NH^3^. As spatial-temporal patterns of daytime and nighttime warming interact with extant seasonal hydrology, energy, and phenology cycles, stark differences in the vegetation responses are generated. However, such observed vegetation growth effects due to seasonal asymmetry in the interannual variations in the mean daytime and nighttime climate (VDNC)^7^,^8^,^9^ and the underlying mechanisms that cause these effects to vary spatially remain poorly understood. Therefore, the effects of seasonal asymmetry in interannual variations in the mean diurnal climate on vegetation activity in the NH need to be better understood, especially because this asymmetry is predicted to increase^10^.

Asymmetry in the seasonal VDNC^11^,^12^, together with other factors (e.g., land use changes^13^), could dramatically modify the seasonal thermo-hydrological patterns that govern land surface-climate feedbacks^14^,^15^. At middle and high latitudes in the NH, this asymmetry could hypothetically even modify the sign of biosphere-atmosphere feedbacks, which are known to be globally significant^15^,^16^,^17^. Therefore, in this study, we quantified the general patterns in the interannual responses of active vegetation growth (AVG, i.e., vegetation growth during the growing season) (for more detail, see Methods) at middle and high northern latitudes (>30°N) using 1) the satellite-derived Normalized Difference Vegetation Index (NDVI)^18^,^19^ and the Fraction of Absorbed Photosynthetically Active Radiation (FAPAR)^20^, 2) FLUXNET-based up-scaled gross primary productivity (GPP)^21^, and 3) tree growth increments to the interannual variability (IAV) of seasonal mean maximum temperature (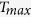), mean minimum temperature (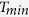) and water availability index (WAI) during past decades (for more detail, see Methods).

## Results

### Covariance between vegetation activity and the seasonal mean 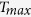


**Figure 1:**
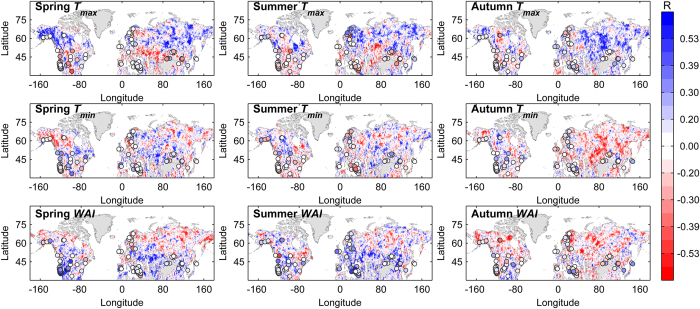
Spatial patterns of the interannual responses of the mean growing-season (April-October) NDVI and TRI to seasonal VDNC in the mid- and high-latitude NH. Spearman partial correlation coefficients between the mean growing-season NDVI (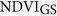) (during 1982–2008) and TRI (dots) (during 1950–2008, if available) as well as between the seasonal mean maximum temperature (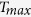), mean minimum temperature (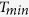) and water availability index (*WAI*) from spring to autumn are shown. *R* = ±0.39, *R* = ±0.30, and *R* = ±0.20 in the colorbar correspond to the 5%, 10% and 20% significance levels for the Spearman partial correlation between interannual variations of 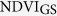 and seasonal mean climate, respectively. This figure is created by MATLAB (R2012b).

**Figure 2:**
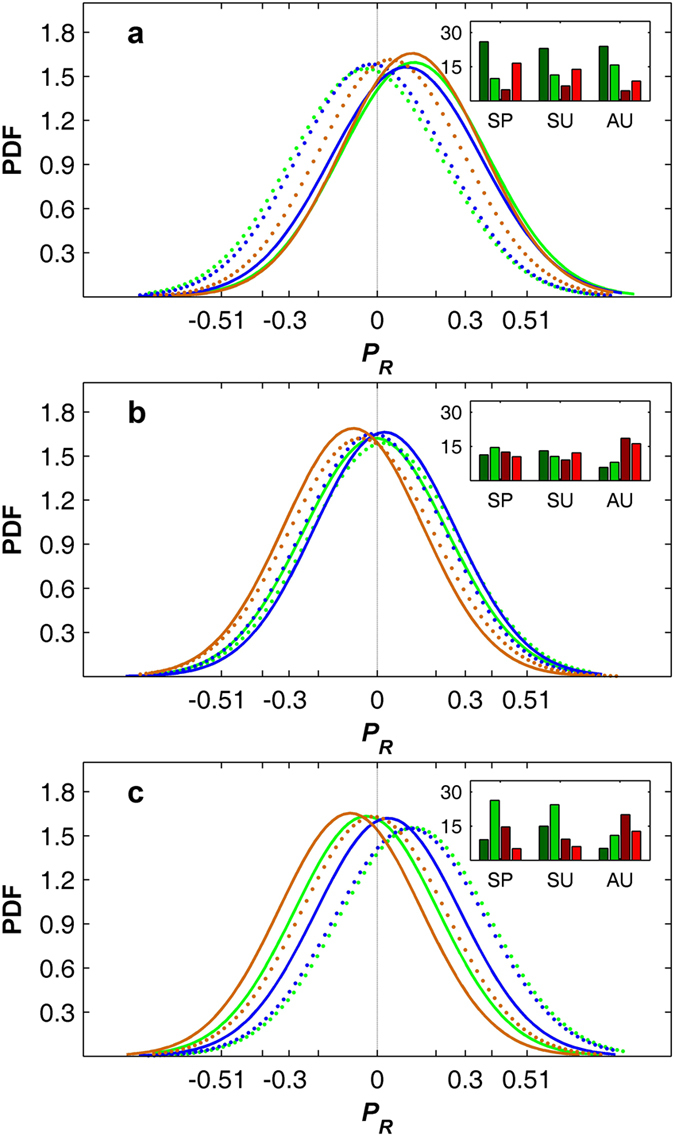
PDFs of the partial correlation coefficients between the mean growing-season (April-October) NDVI and the seasonal VDNC. PDFs of the partial correlation coefficients (*P*_*R*_) between the mean growing-season NDVI and spring (green lines), summer (blue lines) and autumn (brown lines) maximum temperature (**a**), minimum temperature (**b**) and water availability index (**c**) in temperate (<50 °N, dotted lines) and boreal (≥50 °N, solid lines) regions of the NH. The percentages of the pixels that exhibit significant (*p* < 0.10) positive (green bars) and negative (red bars) correlations between the mean growing-season NDVI and spring (SP), summer (SU) and autumn (AU) maximum temperature, minimum temperature and WAI in temperate (light color) and boreal (dark color) regions are specified in the insets. The Spearman correlation coefficients *R* ± 0.51 and ±0.30 in the *x* axis tick label correspond to the 1% and 10% significance levels of Student’s t-test, respectively.

**Figure 3 f3:**
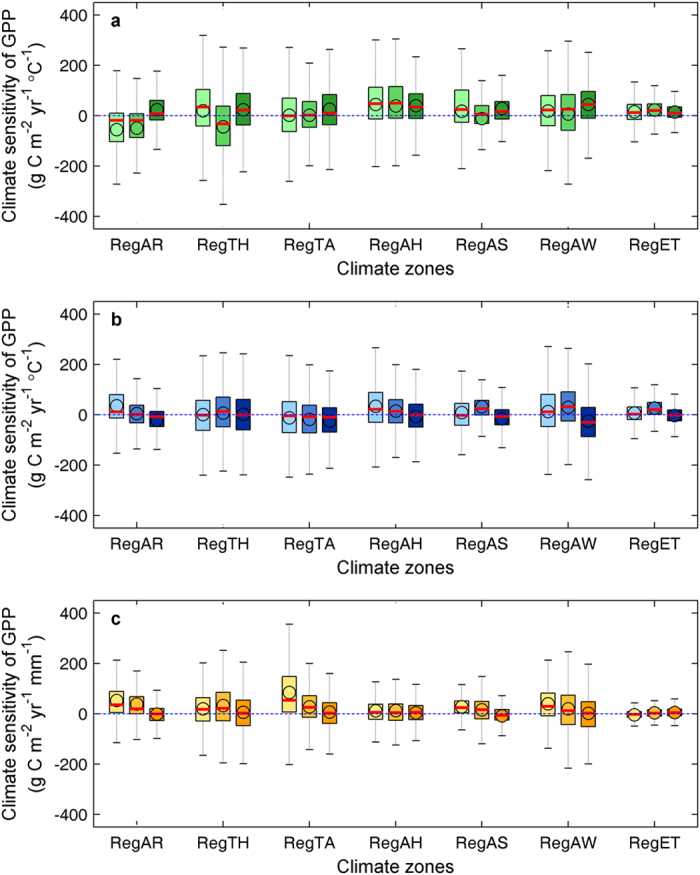
Interannual sensitivity of the growing-season (April-October) GPP to the seasonal VDNC in different climate zones. The sensitivity of the growing-season GPP to spring (light color), summer (medium color) and autumn (dark color) maximum temperature, minimum temperature and WAI in different climate zones are shown in panels a-c, respectively. The circles and short red lines in each box indicate the mean and median values, respectively, of the sensitivity of the growing-season GPP to the seasonal VDNC. The dashed lines and filled areas in each box represent the 5th to 95th and 25th to75th percentiles, respectively, of the sensitivity of the growing-season GPP to the seasonal VDNC. Seven major climate zones based on the Köppen–Geiger climate classification were considered in this study: RegAR (arid region, grouping of BWk, BWh, BSk, BSh), RegTH (temperate humid region, grouping of Cfa, Cfb, and Cfc), RegTA (temperate dry region, grouping of Csa, Csb, Csc, Cwa, Cwb and Cwc), RegAH (cold humid region, grouping of Dfa, Dfb, Dfc, and Dfd), RegAS (cold summer dry region, grouping of Dsa, Dsb, Dsc, and, Dsd), RegAW (cold winter dry region, grouping of Dwa, Dwb, Dwc, and, Dwd), and RegET (polar tundra, ET).

Partial correlation analyses reveal a clear spatial pattern in the interannual responses of the mean growing-season (April-October) NDVI (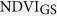) to the IAV of the seasonal mean 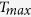 during 1982–2008 (Fig. 1). 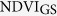 generally exhibits a positive correlation with 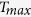 in spring (April-May) (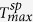), summer (June-August) (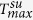) and autumn (September-October) 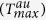 in most (~60–70%) of the boreal NH (Figs 1 and 2a, Supplementary Table S1, Figs S1–S3), with statistically significant (*p* < 0.10) partial correlation coefficients (*R* > 0.30) in ~22–25% of boreal NH despite the great spatial variations in the responses (Fig. 1, Supplementary Table S2 and Figs S1, S3). Such response patterns of the AVG to the IAV of the seasonal mean 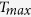 in the boreal NH are also supported by the analyses of mean growing-season FAPAR (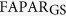) (Supplementary Figs S4a–c and S5a) and the results of independent ridge regression analyses (for more detail, see the supplementary text) of the relationships among 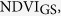 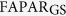 and the seasonal mean 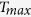 during 1982–2008 (Supplementary Figs S6a–c and S7a–c). Coincidently, the ridge regression analyses reveal a generally positive interannual sensitivity of total growing-season GPP (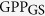) to seasonal mean 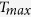 (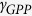, indicated by the coefficient of ridge regression) from spring to autumn in most (~51–89%) of the boreal NH (with statistical significance in ~20–62% of the boreal NH) despite large spatial variations (Fig. 3a, Supplementary Fig. S8a–c), with 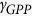 ranging from 36.5 ± 62.4 to 51.3 ± 88.7 g C m^−2^ yr^−1^ °C^−1^ between seasons (Fig. 3a, Supplementary Fig. S8a–c).

By contrast, 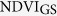 exhibits a significant (*p* < 0.10) negative correlation (*R* < −0.30) with the IAV of mean 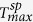 and 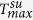 in ~13–16% of the temperate NH (<50 °N) during 1982–2008 (Fig. 1, Supplementary Table S1 and Fig. S1). The most prominent negative correlations are observed in the arid climate zones of the northern U.S.A., southern Eurasia and parts of the Mediterranean regions (Fig. 1, Supplementary Table S2 and Fig. S1), which are generally dominated by temperate grasslands, dry shrublands/forests or forest-grassland ecotones. Analyses of the relationships between the standard tree ring index (TRI) (during 1950–2008, if available), 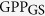, 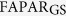 and the seasonal mean 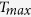 confirm this pattern (Figs 1 and 3a, Supplementary Table S4, Figs S1, S4a–c, S5a, S7 and S8a–c). Partial correlation analyses reveal a generally negative correlation of TRI with mean 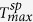 and 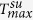 in the temperate NH during 1950–2008 (if available) (with statistical significance for ~15% of samples) compared with a generally positive correlation in the boreal NH (with statistical significance for ~13% of samples) (Fig. 1). Similarly, ridge regression reveals that 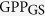 in arid regions of the temperate NH (i.e., RegAR) exhibits a pervasively (~83–85% of RegAR) negative sensitivity to the IAV in 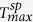 and 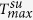 during 1982–2008, with mean 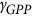 values of –24.9 ± 29.7 and –24.33 ± 28.5 g C m^−2^ yr^−1^ °C^−1^, respectively (Fig. 3a, Supplementary Table S3).

Both partial correlation and ridge regression analyses of the relationships among 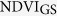, 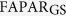, 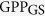, TRI and the seasonal mean 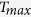 consistently reveal a divergent response of the AVG to the IAV in the seasonal mean 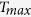 for portions of the middle and high latitudes of the NH in two aspects. First, the AVG in parts of the boreal NH, especially regions with dry summer (i.e., RegAS) and warm temperate regions (e.g., the western and southeastern U.S.A.), responds differently to mean 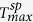 and 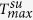 (Fig. 1, Supplementary Table S2, Table S3, Figs S1, S3, S4a,b and S6-S7a,b): The pervasive (~31%) significant positive correlation (*R* > 0.30) between 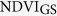 and 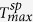 in RegAS tends to weaken (~9.1%) in summer (Fig. 1, Supplementary Table S2, Figs S1 and S3), whereas the 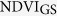 maintains a significant positive correlation (*R* > 0.30) with the WAI (for ~15–17% of the samples) in both spring and summer (Fig. 1, Supplementary Table S2, Figs S1 and S2c). Eddy covariance observations in these regions support this notion of seasonal water limitation, indicating that 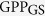 responds positively to WAI in spring and summer during 1982–2008 (with spring and summer 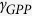 values of 1.2 ± 1.7 and 0.9 ± 1.8 g C m^−2^ yr^−1^ mm^−1^, respectively, in RegAS) (Fig. 3c, Supplementary Table S3, Figs S8g,h,S9g,h and S10). In addition to the regions with dry summers in the boreal NH, 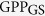 in certain parts of the temperate NH also exhibits divergent interannual sensitivity to 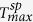 and 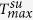 (Fig. 3a, S9a,b and S10a), with 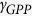 values of 8.9 ± 22.1 in spring and −20.9 ± 43.6 g C m^−2^ yr^−1^ °C^−1^ in summer in RegTH (Fig. 3a, Supplementary Fig. S8a,b). At the same time, the TRI values in the temperate NH exhibit pervasive positive correlations with 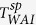 and 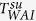 (with statistical significance at *p* < 0.05 level in ~14% and ~40% of samples, respectively) during 1950–2008 (Figs 1 and S1). Second, the interannual responses of the AVG to 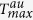 diverge from the responses to 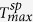 and 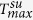 in the majority of the temperate NH, especially in regions with drought limitations (e.g., RegAR and RegTA) (Supplementary Table S2). The 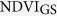 exhibits a significant positive correlation (*R* > 0.30) with mean 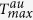 in ~15–16% of the temperate NH but exhibits a significant positive correlation with 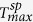 and 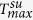 in only ~<8% of that area (Figs 1 and 2a, Table S2, Fig. S1 and S2a).

### Covariance between vegetation activity and the seasonal mean 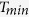


The interannual responses of the AVG in the middle and high latitudes of the NH to the mean spring and summer minimum temperatures (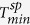 and 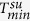, respectively) are relatively ambiguous (Fig. 1, Supplementary Fig. S1, S2b, S4d,e, S5b and S6-S9d,e), yet the trends in 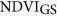, 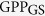 and TRI indicate that the AVG responds positively to 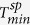 in certain parts of the temperate NH, particularly in arid regions (primarily in the central U.S.A. and southern Eurasia). The correlation with 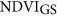 is statistically significant (*R* > 0.30, *p* < 0.10) in ~17.5% of these regions (Fig. 1, Supplementary Table S2, Fig. S1). Interestingly, such positive responses of the AVG to mean 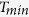 tend to weaken from spring to summer in arid and temperate arid regions (Figs 1 and 3d,e, Supplementary Fig. S1) and instead become predominant in the high-latitude NH. Significant positive responses of 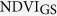 and 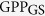 to 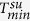 are observed in ~12–20% and ~23–64%, respectively, of cold regions and tundra (RegAH, RegAS and RegET) (Figs 1 and 3b, Supplementary Fig. S1, S3 and Table S2). Coincidently, the TRI exhibits a more pervasive positive correlation with 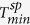 (~15.8%) than with 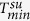 (~7.9%) in the temperate NH (Fig. 1).

Contrary to the ambiguous relationships between AVG and 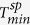 and 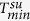, both 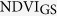 and 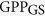 exhibit pervasively (60–63%) negative responses to 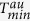 in most of the temperate and cold NH except in certain mountainous regions (e.g., the Rocky Mountains in western North America). Statistical significance is observed for 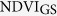 in ~16% and ~18% of the temperate and boreal NH, respectively (Figs 1,2b and 3b[Fig f3] Supplementary Table S1, Table S3, Fig. S1 and Fig. S3). However, TRI data indicate a persistently positive rather than negative correlation with 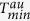 during 1950–2008 (Fig. 1, Supplementary Fig. S1). Additionally, the observed divergent response patterns between AVG and seasonal VDNC are not susceptible to the arbitrary definitions of active growing seasons (Supplementary Fig. S11-S12) and selection of analysis periods (see Methods for details) (Supplementary Fig. S13).

## Discussion

**Figure 4:**
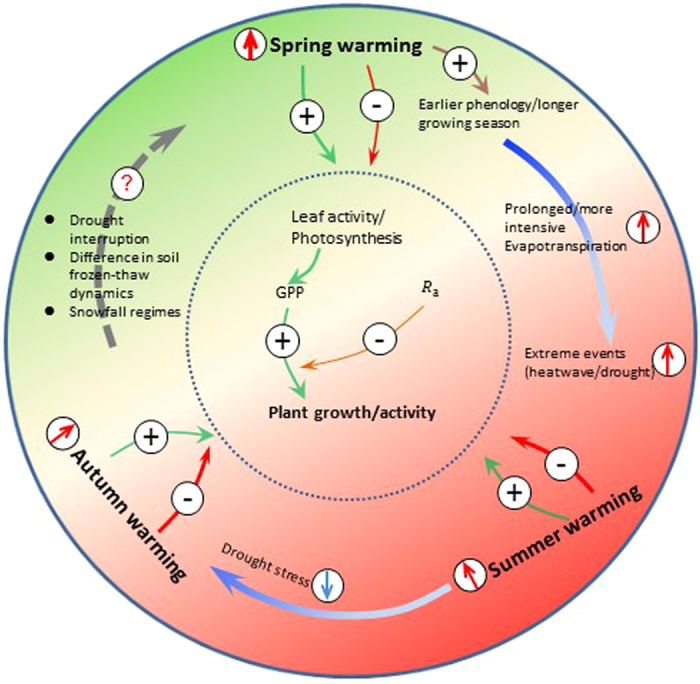
Conceptual diagram for water-mediated interannual responses of vegetation activity to the seasonal VDNC in the mid- and high-latitudinal NH. The green and red lines indicate the effects of seasonal variations in diurnal warming on plant photosynthesis/GPP and autotrophic respiration (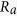), respectively. The relative importance of the impacts and the relative warming rates between seasons are indicated by the line width (broader lines indicate greater importance). The blue arrow indicates the general soil water content following the transitions of seasons. The lighter blue color indicates more intense drought limitations for vegetation growth. The gray arrow indicates an unclear process. See also Reichstein *et al*. (2013) for the effects of extreme events on terrestrial ecosystems.

The results of both the partial correlation and ridge regression analyses demonstrate that the AVG in the boreal NH is generally temperature-limited: a warmer mean daytime temperature (i.e., 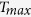) during the active growing season can intuitively enhance the vegetation activity^1^,^2^,^4^,^22^,^23^. However, we have identified an obvious seasonally divergent response of the AVG to the IAV in seasonal mean 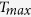 in parts of the high-latitude NH, especially in regions with dry summers. The AVG responds differently to 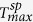 and 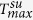 (Fig. 1, Supplementary Table S2, Figs S1, S3, S4a,b and S5-S7a,b), and a pervasive (~31%) significant positive correlation is observed between 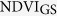 and 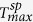 shading in summer (Fig. 1, Supplementary Table S2 and Fig. S3). In fact, the AVG in cold regions with dry summers is vulnerable to water stress under daytime warming, thereby resulting in seasonal drought^16^,^24^,^25^, as confirmed by the close (~65.8–66%) positive coupling between the IAV in 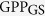 and the WAI during spring and summer. In addition to regions with dry summers in the boreal NH, the AVG in certain parts of the temperate NH also responds differently to mean 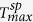 and 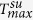 (Fig. 1 Supplementary Figs S3, S6-S9a,b and S10a). The observed divergent responses of the AVG to 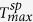 and 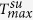 in these regions may be partially attributed to the daytime warming-induced progressive drought from spring to summer, as revealed by a water balance equation and the relationships between the seasonal soil moisture and the VDNC during 1982–2008 (Fig. 4, Supplementary Figs S14-S16). TRI data reveal that tree growth in the temperate NH experiences continuous water limitations during spring and summer, as revealed by pervasive positive correlations of the TRI with 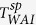 and 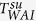. In fact, warmer daytime temperatures in spring can lead to an earlier spring phenology and greater photosynthesis^26^,^27^, thereby enhancing vegetation growth as long as the soil water and atmospheric vapor pressure are sufficient. Conversely, if the increased warming rates during the early growing season are unmatched by water resources, the warmer temperatures can trigger a prolonged warming-induced drought, especially in regions that suffer seasonal drought limitations (e.g., RegAS and most regions in the temperate NH) (Fig. 4). Such cases of excessive soil water stress or, more commonly, vapor pressure deficits (Supplementary Figs S14-S16, ref. Liu *et al*., 2013^28^) can dramatically reduce vegetation growth and even negate springtime ecosystem carbon gains^16^,^25^,^29^.

In addition to the divergent responses of the AVG to mean 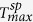 and 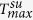 in parts of the mid- and high-latitude NH, the AVG responds differently to 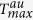 than to 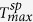 and 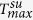 across most of the temperate NH. Significant positive correlations between AVG and 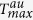 are observed in nearly twice as many areas as those of 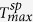 and 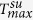. Early and mid-growing season (e.g., spring and early summer) warming tends to trigger regional droughts (Supplementary Fig. S14-S16), which can exert strong control on vegetation growth and even cause tree mortality in the temperate NH and some parts of the cold NH^28^,^30^,^31^,^32^. Nevertheless, the emergence of a positive response of 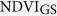 to 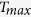 in autumn may reflect alleviation of water stress on photosynthetic activity in that period (Figs 1 and 4, Supplementary Figs S1, S14)^33^, which is confirmed by a generally negative correlation between 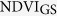 and the autumn WAI (Fig. 1). Consequently, alleviation of water limitations in autumn (Fig. 4, Supplementary Fig. S14) results in a generally positive correlation between the TRI and 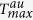 in ~49% of the temperate NH except in portions of arid regions (e.g., the central U.S.A.). By contrast, a predominantly (~60–70%) negative correlation exists between the TRI and 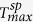 and 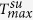 (Fig. 1, Supplementary Figs S1, S13). These factors also lead to a conversion from a generally (~55–81%) negative correlation between 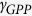 and 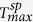 and 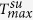 to a positive correlation (in ~57–67% of the temperate NH) between 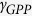 and 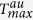 in the temperate NH (Supplementary Fig. S8a–c).

The positive responses of the AVG to variations in the mean nighttime temperatures 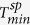 and 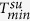 in parts of NH are most likely attributable to the reported compensation effects of nocturnal warming^34^. However, the shifting responses of the AVG to 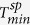 and 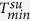 in arid and temperate arid regions (Fig. 1, Supplementary Fig. S1, S3 and Table S2) imply that the compensation effects of nocturnal warming on vegetation growth might be seasonally and spatially dependent and are likely modified by other bio-physical factors, such as water conditions (Fig. 4) and plant functional types^35^. In contrast to the ambiguous relationships between the AVG and 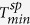 and 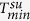, there is a pervasive negative response of the AVG, from the perspective of 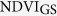 and 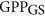, to 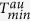 across most of the temperate and cold NH except in certain mountainous regions (e.g., the Rocky Mountains in western North America). The dominant effect of autumn nocturnal warming on NH vegetation growth may be enhanced plant respiration, which consequently leads to autumn carbon loss^36^. However, the TRI data indicate a persistent positive rather than negative correlation with 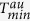 (Fig. 1). In addition to enhanced whole-plant respiration, warmer autumn (both day and night) temperatures may reactivate cambial activity^37^. Combined with alleviation of water limitations (Fig. 4, Supplementary Figs S14-S17), these process may partially explain the observed widespread positive correlation between the TRI and 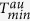 in the temperate NH. Overall, the observed divergent responses of AVG to seasonal 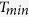 in portions of the NH are to some degree attributable to the water-mediated counterbalancing effects of 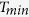 on vegetation growth and respiration^34^,^38^,^39^.

In summary, remote sensing retrievals, empirical up-scaling measurements from FLUXNET, and tree growth data consistently indicate a possible water-mediated divergent response of vegetation activity to seasonal VDNC, especially in temperate regions and boreal regions with dry summers in the NH. Our research provides new insights into the seasonally divergent responses of the AVG to the seasonal asymmetry in the VDNC and reinforces the idea that the projected intensification in the asymmetry in the seasonal VDNC under continued global warming and increasing climate extremes^40^ may have strong, spatially variable effects on vegetation growth and carbon dynamics in the NH, with associated changes in regional ecosystem services, the terrestrial carbon cycle, and global climate feedbacks.

## Methods

### Remote sensing data and up-scaled GPP

We used three types of data to investigate how AVG (i.e., vegetation growth during the growing season) responded to the mean seasonal VDNC: the satellite-derived AVHRR/GIMMS Normalized Difference Vegetation Index (NDVI, specifically version NDVI3g)^18^,^19^, the satellite-derived Fraction of Absorbed Photosynthetically Active Radiation (FAPAR)^20^, and FLUXNET-based up-scaled gross primary production (GPP) data^21^. The data featured a spatial resolution of 0.5° × 0.5° and a monthly temporal resolution for 1982–2008. Pixels with a multi-year mean annual NDVI value <0.10 were masked in our study.

### Tree ring data

In total, 149 standard TRI chronologies from the mid- and high-latitude NH were used to investigate the interannual responses of tree growth to the seasonal VDNC. Standard TRI chronologies were built following the standard procedures of dendrochronology^41^, including fitting a curve to the raw ring-width series and then dividing each ring-width value by the corresponding curve value to generate a series of growth indices. Of these chronologies, 136 were obtained from the International Tree-Ring Data Bank (ITRDB, http://www.ncdc.noaa.gov/data-access/paleoclimatology-data/datasets/tree-ring) based on four criteria: 1) intact records of latitude, longitude, elevation, species and sample depth; 2) the chronologies cover at least 1950–1998 or extend to more recent times (i.e., early 21st century); 3) the sample depth for each site-year is greater than 10; and 4) the altitude of the sample site is less than the 90^th^ percentile of the elevation distribution of the surrounding 0.5° × 0.5° region based on the GTOPO30 dataset (with ~1 km resolution, available from U.S. Geological Survey, https://lta.cr.usgs.gov/GTOPO30) to match the regional vegetation growth patterns and avoid site-specific representations. Our research group provided another 13 standard chronologies from northern China. Our chronologies satisfy the four criteria listed above (see Table S4 for details).

### Climate data

Monthly mean maximum (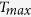, averaging daily maximum temperature) and mean minimum temperature (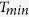, averaging daily minimum temperature) at a spatial resolution of 0.5° were obtained from the newly updated CRU TS 3.22 dataset (http://www.cru.uea.ac.uk/). The seasonal (in detail see section of sensitivity test) mean 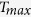 and 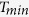 were then calculated by averaging the monthly mean climate data. The seasonal mean 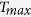 and 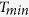 are used in this study to represent the general climate conditions of seasonal mean daytime and nighttime temperature^3^, respectively. Remotely sensed soil moisture data with a daily resolution for the period 1978–2013 were obtained from the Essential Climate Variable Soil Moisture dataset (ECV SM 02.0) (http://www.esa-soilmoisture-cci.org/). These data were produced following the method described by Liu *et al*. (2011) and Liu *et al*. (2012)^42^,^43^, representing surface soil moisture with a global coverage and spatial resolution of 0.25°. We then resampled this surface soil moisture data to a spatial resolution of 0.5° × 0.5°. Unfortunately, the soil moisture data were not continuous during 1982–2008, and only pixels with at least 19 years of data between 1982 and 2008 were retained.

### Water deficit and WAI

The monthly water deficit was estimated using a simple water balance equation:


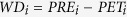
 where 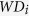, 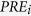, and 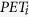 are the water deficit (positive values indicate water surpluses), precipitation and potential evapotranspiration in month *i*. The potential evapotranspiration is calculated using Thornthwaite’s equation^44^. Then, the total seasonal water deficit is calculated by summing the monthly *WD* values. Negative values express the degree of dryness; the more negative the value, the more intense the water limitation. In our study, the monthly *WAI* was calculated based on the water balance equation proposed by Kleidon and Heimann (1998)^45^ and was used to represent the soil moisture conditions.

### Statistical analysis

Spearman partial correlation and ridge regression were performed to investigate the responses of vegetation activity in the mid- and high-latitude NH to the mean seasonal VDNC. The interannual sensitivity of vegetation growth to IAV in the seasonal VDNC was evaluated using the ridge regression coefficients. Ridge regression has been demonstrated to be an effective method for addressing collinearity in multivariate regression^46^. The probability density function (PDF) of the Spearman partial correlation coefficients between the seasonal VDNC and the IAV in the AVG was estimated for seven major climate zones (as defined by the Köppen–Geiger climate classification^47^), including arid (RegAR, grouping of BWk, BWh, BSk, and BSh), warm temperate humid (RegTH, grouping of Cfa, Cfb and Cfc), warm temperate arid (RegTA, grouping of Csa, Csb, Csc, Cwa, Cwb, and Cwc), cold humid (RegAH, grouping of Dfa, Dfb, Dfc, and Dfd), cold summer dry (RegAS, grouping of Dsa, Dsb, Dsc, and Dsd), cold winter dry (RegAW, grouping of Dwa, Dwb, Dwc, Dwd) and polar tundra (RegET). Prior to performing the Spearman partial correlation and ridge regression analyses, all variables (i.e., NDVI, FAPAR, GPP, TRI and the climate variables) were detrended using a linear function. The statistical analyses and figure creation were performed using the MATLAB software package (R2012b).

### Sensitivity test

In this study, we attempted to define the seasons (spring, summer, and autumn) in a manner that is consistent across the mid- and high-latitude NH. Notably, three different definitions of seasons were used in our study to verify the robustness of our conclusions relative to the arbitrary definitions of vegetation growth seasons in the mid- and high-latitude NH. The three definitions are as follows:

Definition 1: spring: April-May; summer: June-August; autumn: September-October

Definition 2: spring: April-May; summer: June-August; autumn: September

Definition 3: spring: May; summer: June-August; autumn: September-October

We then performed the same analyses for the three different season definitions. The results of Definition 1 are presented in the main text, and the results of the other two definitions are presented in the supplementary materials.

## Additional Information

**How to cite this article**: Wu, X. *et al*. Seasonal divergence in the interannual responses of Northern Hemisphere vegetation activity to variations in diurnal climate. *Sci. Rep*. **6**, 19000; doi: 10.1038/srep19000 (2016).

## Supplementary Material

Supplementary Information
