# Supplementary material for: Seasonal divergence in the interannual responses of Northern Hemisphere vegetation activity to variations in diurnal climate
Source: Sci Rep. 2016 Jan 11;6:19000. doi: 10.1038/srep19000 (PMC4707447; doi:10.1038/srep19000)
Supplement: Supplementary Information [file srep19000-s1.doc]

Supplementary materials for

**Seasonal divergence in the interannual responses of Northern Hemisphere vegetation activity to variations in diurnal climate**

Xiuchen Wu^1, 2, 3*^, Hongyan Liu^4^, Xiaoyan Li^1, 2, 3^, Eryuan Liang^5^, Pieter S.A. Beck^6^, Yongmei Huang^1, 3^

1. State Key Laboratory of Earth Processes and Resource Ecology, Beijing Normal University, Beijing, 100875, China
2. Joint Center for Global Change Studies, Beijing, 100875, China
3. College of Resources Science and Technology, Beijing Normal University, Beijing, 100875, China
4. College of Urban and Environmental Science, Peking University, Beijing, 100871, China
5. Key Laboratory of Tibetan Environment Changes and Land Surface Processes, Institute of Tibetan Plateau Research, Chinese Academy of Sciences, Beijing, 100085, China
6. Forest Resources and Climate Unit, Institute for Environment and Sustainability (IES), Joint Research Centre (JRC), European Commission, Ispra, VA, Italy

Correspondence: [xiuchen.wu@bnu.edu.cn](mailto:xiuchen.wu@bnu.edu.cn), Phone, 0086-10-58807656, Fax, 0086-10-58807656

This file contains:

1. Table S1-S4

2. Figure S1-S17

3. References

**Table S1**. Percentages of pixels showing positive and negative Spearman correlations between mean growing-season (April-October) NDVI and seasonal mean climate during 1982-2008 in temperate and boreal northern hemisphere.

| Climate Variables | Temperate NH (°30N-°50N) | | | | | |  | Boreal NH (>50°N) | | | | | |
| --- | --- | --- | --- | --- | --- | --- | --- | --- | --- | --- | --- | --- | --- |
|  | %+ | %10+ | %5+ | %5- | %10- | %- |  | %+ | %10+ | %5+ | %5- | %10- | %- |
| 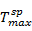 | 45.3 | 9.3 | 4.5 | 9.3 | 15.8 | 54.7 |  | 68.8 | 25.0 | 15.5 | 2.0 | 4.5 | 31.2 |
| 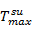 | 46.9 | 10.9 | 5.6 | 7.1 | 13.1 | 53.1 |  | 65.3 | 22.1 | 13.3 | 3.1 | 6.2 | 34.7 |
| 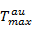 | 57.4 | 15.0 | 8.1 | 4.4 | 8.2 | 42.6 |  | 68.7 | 22.9 | 13.5 | 1.8 | 4.1 | 31.3 |
| 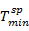 | 52.5 | 13.9 | 7.5 | 5.0 | 10.0 | 47.5 |  | 49.4 | 10.6 | 5.2 | 6.2 | 11.8 | 50.6 |
| 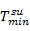 | 47.4 | 10.1 | 5.1 | 5.9 | 11.6 | 52.6 |  | 54.4 | 12.5 | 6.5 | 4.3 | 8.6 | 45.6 |
| 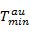 | 40.8 | 7.6 | 3.8 | 8.8 | 15.5 | 59.2 |  | 36.6 | 5.4 | 2.5 | 9.6 | 17.5 | 63.4 |
| 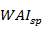 | 68.2 | 25.3 | 16.2 | 2.3 | 4.8 | 31.8 |  | 43.4 | 8.6 | 4.2 | 7.1 | 13.9 | 56.6 |
| 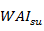 | 67.0 | 23.6 | 14.4 | 2.8 | 5.6 | 33.0 |  | 56.1 | 14.1 | 7.4 | 4.3 | 8.6 | 43.9 |
| 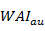 | 47.7 | 10.4 | 5.3 | 6.2 | 12.1 | 52.3 |  | 36.1 | 4.9 | 1.9 | 11.2 | 19.0 | 63.9 |

Note: %+ and %– indicate percentages of pixels showing positive and negative correlations between mean growing-season (April-October) NDVI and seasonal mean climate. %10 and %5 indicate percentages of pixels showing significant correlations between mean growing-season (April-October) NDVI and seasonal mean climate at 0.1 and 0.05 levels, respectively. *R* = ±0.39 and *R* = ±0.30 correspond to the 5% and 10% significance levels for the Spearman partial correlation between interannual variations of mean growing-season NDVI and seasonal mean climate, respectively.

**Table S2**. Same as Table S1 but for different climate zones.

| Climate Variables | RegAR | | | | | |  | RegTH | | | | | |  | RegTA | | | | | |
| --- | --- | --- | --- | --- | --- | --- | --- | --- | --- | --- | --- | --- | --- | --- | --- | --- | --- | --- | --- | --- |
|  | %+ | %10+ | %5+ | %5- | %10- | %- |  | %+ | %10+ | %5+ | %5- | %10- | %- |  | %+ | %10+ | %5+ | %5- | %10- | %- |
| 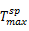 | 38.4 | 7.0 | 3.4 | 13.6 | 21.8 | 61.6 |  | 44.9 | 7.6 | 3.6 | 6.2 | 11.6 | 55.1 |  | 48.6 | 8.7 | 4.4 | 7.6 | 14.3 | 51.4 |
| 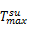 | 39.9 | 7.8 | 3.8 | 9.7 | 17.4 | 60.1 |  | 47.9 | 12.0 | 6.3 | 7.1 | 13.2 | 52.1 |  | 54.5 | 11.8 | 5.7 | 3.5 | 7.4 | 45.5 |
| 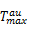 | 57.9 | 15.8 | 9.0 | 4.4 | 8.2 | 42.1 |  | 61.0 | 15.6 | 8.2 | 2.9 | 5.3 | 39.0 |  | 53.5 | 14.7 | 7.8 | 6.5 | 12.3 | 46.5 |
| 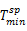 | 54.6 | 17.5 | 9.8 | 4.8 | 9.5 | 45.4 |  | 54.0 | 11.3 | 5.4 | 4.5 | 9.2 | 46.0 |  | 47.1 | 11.4 | 5.5 | 5.8 | 11.6 | 52.9 |
| 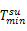 | 53.4 | 12.9 | 7.0 | 5.1 | 10.1 | 46.6 |  | 41.9 | 8.5 | 4.5 | 7.6 | 13.7 | 58.1 |  | 40.5 | 7.4 | 3.5 | 7.2 | 14.4 | 59.5 |
| 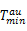 | 39.1 | 6.4 | 2.7 | 11.0 | 17.8 | 60.9 |  | 36.7 | 4.5 | 2.5 | 8.0 | 15.2 | 63.3 |  | 45.4 | 8.9 | 4.4 | 5.7 | 10.6 | 54.6 |
| 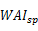 | 78.7 | 34.1 | 23.1 | 0.5 | 1.6 | 21.3 |  | 53.9 | 15.2 | 8.1 | 4.5 | 8.9 | 46.1 |  | 76.8 | 36.3 | 24.5 | 1.2 | 2.3 | 23.2 |
| 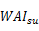 | 73.4 | 28.6 | 18.3 | 1.1 | 2.6 | 26.6 |  | 65.4 | 24.4 | 15.0 | 3.4 | 7.5 | 34.6 |  | 59.3 | 14.8 | 8.0 | 3.6 | 6.5 | 40.7 |
| 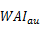 | 44.9 | 8.7 | 4.5 | 6.6 | 12.7 | 55.1 |  | 53.6 | 14.8 | 8.2 | 4.7 | 9.3 | 46.4 |  | 43.7 | 6.1 | 2.4 | 6.3 | 12.1 | 56.3 |

**Table S2**. (Continued)

| Climate Variables | RegAH | | | | | |  | RegAS | | | | | |  | RegAW | | | | | |  | RegET | | | | | |
| --- | --- | --- | --- | --- | --- | --- | --- | --- | --- | --- | --- | --- | --- | --- | --- | --- | --- | --- | --- | --- | --- | --- | --- | --- | --- | --- | --- |
|  | %+ | %10+ | %5+ | %5- | %10- | %- |  | %+ | %10+ | %5+ | %5- | %10- | %- |  | %+ | %10+ | %5+ | %5- | %10- | %- |  | %+ | %10+ | %5+ | %5- | %10- | %- |
| 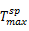 | 66.7 | 23.9 | 14.7 | 2.8 | 5.9 | 33.3 |  | 57.6 | 31.0 | 21.3 | 8.4 | 13.3 | 42.4 |  | 62.3 | 16.4 | 7.3 | 2.9 | 6.8 | 37.7 |  | 63.7 | 20.2 | 12.5 | 3.5 | 5.7 | 36.3 |
| 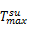 | 61.9 | 20.0 | 11.9 | 3.8 | 7.4 | 38.1 |  | 41.9 | 9.1 | 4.0 | 7.4 | 13.5 | 58.1 |  | 64.8 | 23.4 | 13.6 | 3.7 | 7.2 | 35.2 |  | 68.7 | 22.8 | 13.6 | 1.6 | 3.8 | 31.3 |
| 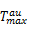 | 66.9 | 22.1 | 13.1 | 2.4 | 5.1 | 33.1 |  | 60.1 | 19.4 | 9.5 | 4.8 | 9.1 | 39.9 |  | 68.2 | 22.2 | 12.2 | 1.2 | 3.4 | 31.8 |  | 63.6 | 16.4 | 8.7 | 1.9 | 4.4 | 36.4 |
| 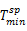 | 50.3 | 11.5 | 5.9 | 5.6 | 11.2 | 49.7 |  | 43.9 | 13.5 | 8.4 | 15.8 | 21.7 | 56.1 |  | 49.9 | 9.8 | 4.8 | 4.8 | 9.9 | 50.1 |  | 47.8 | 9.2 | 4.4 | 7.3 | 13.3 | 52.2 |
| 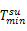 | 52.3 | 11.7 | 6.0 | 4.7 | 9.4 | 47.7 |  | 62.0 | 20.3 | 11.8 | 3.2 | 6.1 | 38.0 |  | 49.7 | 7.9 | 3.4 | 4.1 | 9.2 | 50.3 |  | 59.9 | 15.1 | 8.0 | 3.1 | 6.4 | 40.1 |
| 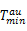 | 37.3 | 5.8 | 2.8 | 9.7 | 17.4 | 62.7 |  | 45.6 | 10.8 | 6.1 | 8.2 | 15.4 | 54.4 |  | 31.6 | 7.1 | 3.6 | 11.2 | 22.0 | 68.4 |  | 44.2 | 7.3 | 3.5 | 6.3 | 12.2 | 55.8 |
| 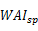 | 47.3 | 10.5 | 5.6 | 6.1 | 12.2 | 52.7 |  | 65.3 | 15.2 | 8.5 | 3.6 | 6.3 | 34.7 |  | 59.5 | 16.8 | 9.3 | 2.6 | 6.6 | 40.5 |  | 34.2 | 5.1 | 2.4 | 11.1 | 20.1 | 65.8 |
| 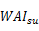 | 56.4 | 14.9 | 7.9 | 4.4 | 8.6 | 43.6 |  | 57.9 | 16.7 | 12.0 | 6.1 | 12.0 | 42.1 |  | 65.1 | 22.8 | 13.4 | 3.4 | 6.5 | 34.9 |  | 59.3 | 12.7 | 6.0 | 3.3 | 7.3 | 40.7 |
| 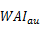 | 37.8 | 5.8 | 2.4 | 10.6 | 18.0 | 62.2 |  | 39.8 | 3.8 | 0.8 | 10.1 | 17.3 | 60.2 |  | 40.0 | 7.9 | 3.3 | 9.8 | 17.8 | 60.0 |  | 38.4 | 5.2 | 2.2 | 10.1 | 18.7 | 61.6 |

Note: %+ and %– indicate percentages of pixels showing positive and negative correlations between mean growing-season (April-October) NDVI and seasonal mean climate. %10 and %5 indicate percentages of pixels showing significant correlations between mean growing-season (April-October) NDVI and seasonal mean climate at 0.1 and 0.05 level, respectively. *R* = ±0.39 and *R* = ±0.30 correspond to the 5% and 10% significance levels for the Spearman partial correlation between interannual variations of mean growing-season NDVI and seasonal mean climate, respectively. Seven major climate zones based on Köppen–Geiger climate classification are considered in this study: RegAR (arid region, grouping of BWk, BWh, BSk, BSh), RegTH (temperate humid region, grouping of Cfa, Cfb, and Cfc), RegTA (temperate dry region, grouping of Csa, Csb, Csc, Cwa, Cwb and Cwc), RegAH (cold humid region, grouping of Dfa, Dfb, Dfc, and Dfd), RegAS (cold summer dry region, grouping of Dsa, Dsb, Dsc, and, Dsd), RegAW (cold winter dry region, grouping of Dwa, Dwb, Dwc, and, Dwd), and RegET (polar tundra, ET).

**Table S3**. Percentages of pixels showing positive and negative interannual sensitivity of growing-season (April-October) GPP to seasonal climate in different climate zones.

| Climate Variables | RegAR | | | | | |  | RegTH | | | | | |  | RegTA | | | | | |
| --- | --- | --- | --- | --- | --- | --- | --- | --- | --- | --- | --- | --- | --- | --- | --- | --- | --- | --- | --- | --- |
|  | %+ | %10+ | %5+ | %5- | %10- | %- |  | %+ | %10+ | %5+ | %5- | %10- | %- |  | %+ | %10+ | %5+ | %5- | %10- | %- |
| 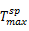 | 15.04 | 0.36 | 0.06 | 25.79 | 34.51 | 84.96 |  | 48.14 | 6.18 | 3.24 | 8.19 | 12.43 | 51.86 |  | 22.08 | 2.17 | 1.47 | 20.06 | 27.32 | 77.92 |
| 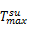 | 17.28 | 0.85 | 0.49 | 20.53 | 28.68 | 82.72 |  | 35.22 | 6.22 | 4.10 | 13.40 | 18.95 | 64.78 |  | 52.13 | 6.71 | 3.77 | 4.89 | 7.20 | 47.87 |
| 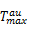 | 56.17 | 11.45 | 6.87 | 3.80 | 6.23 | 43.83 |  | 53.72 | 11.24 | 7.45 | 2.64 | 5.36 | 46.28 |  | 56.81 | 15.44 | 10.27 | 7.48 | 10.55 | 43.19 |
| 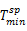 | 44.78 | 4.68 | 2.07 | 3.74 | 6.87 | 55.22 |  | 45.57 | 2.83 | 1.27 | 4.80 | 8.34 | 54.43 |  | 36.06 | 4.12 | 1.61 | 8.32 | 12.72 | 63.94 |
| 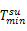 | 42.01 | 3.77 | 1.61 | 5.92 | 9.42 | 57.99 |  | 39.02 | 3.05 | 1.38 | 3.91 | 8.00 | 60.98 |  | 40.18 | 3.91 | 2.45 | 5.87 | 9.85 | 59.82 |
| 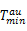 | 31.90 | 2.49 | 1.22 | 10.18 | 16.34 | 68.10 |  | 36.37 | 2.76 | 1.79 | 3.16 | 6.89 | 63.63 |  | 41.79 | 5.03 | 2.94 | 4.54 | 8.39 | 58.21 |
| 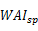 | 92.01 | 51.61 | 41.74 | 0.03 | 0.27 | 7.99 |  | 53.31 | 12.62 | 7.56 | 2.57 | 5.10 | 46.69 |  | 86.65 | 46.19 | 36.90 | 0.14 | 0.56 | 13.35 |
| 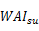 | 87.91 | 38.46 | 29.89 | 0.09 | 0.21 | 12.09 |  | 70.81 | 24.01 | 18.39 | 1.38 | 3.31 | 29.19 |  | 67.85 | 13.91 | 8.87 | 1.26 | 2.66 | 32.15 |
| 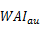 | 46.84 | 6.32 | 3.74 | 3.74 | 6.77 | 53.16 |  | 51.04 | 8.97 | 5.55 | 4.43 | 8.27 | 48.96 |  | 43.05 | 3.00 | 1.33 | 5.38 | 9.85 | 56.95 |

**Table S3**. (Continued)

| Climate Variables | RegAH | | | | | |  | RegAS | | | | | |  | RegAW | | | | | |  | RegET | | | | | |
| --- | --- | --- | --- | --- | --- | --- | --- | --- | --- | --- | --- | --- | --- | --- | --- | --- | --- | --- | --- | --- | --- | --- | --- | --- | --- | --- | --- |
|  | %+ | %10+ | %5+ | %5- | %10- | %- |  | %+ | %10+ | %5+ | %5- | %10- | %- |  | %+ | %10+ | %5+ | %5- | %10- | %- |  | %+ | %10+ | %5+ | %5- | %10- | %- |
| 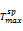 | 83.51 | 44.33 | 35.07 | 2.19 | 3.39 | 16.49 |  | 50.58 | 27.91 | 19.96 | 17.64 | 23.06 | 49.42 |  | 59.68 | 21.27 | 14.92 | 4.59 | 7.78 | 40.32 |  | 80.98 | 32.21 | 24.26 | 0.68 | 1.14 | 19.02 |
| 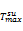 | 75.09 | 31.21 | 23.04 | 6.58 | 8.35 | 24.91 |  | 55.04 | 14.92 | 9.69 | 11.24 | 14.73 | 44.96 |  | 63.35 | 28.92 | 21.41 | 6.90 | 10.33 | 36.65 |  | 91.16 | 46.10 | 33.42 | 0.89 | 1.39 | 8.84 |
| 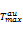 | 73.34 | 20.46 | 13.34 | 0.66 | 1.44 | 26.66 |  | 71.51 | 18.41 | 10.08 | 2.33 | 3.68 | 28.49 |  | 73.54 | 16.03 | 8.85 | 0.14 | 0.42 | 26.46 |  | 73.17 | 14.57 | 8.51 | 0.11 | 0.43 | 26.83 |
| 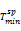 | 82.64 | 30.90 | 22.75 | 0.76 | 1.41 | 17.36 |  | 48.06 | 8.72 | 5.04 | 1.94 | 5.04 | 51.94 |  | 62.33 | 20.62 | 15.20 | 2.13 | 4.22 | 37.67 |  | 73.89 | 20.73 | 13.68 | 0.46 | 0.96 | 26.11 |
| 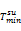 | 69.37 | 16.43 | 10.66 | 1.18 | 2.37 | 30.63 |  | 62.02 | 15.31 | 10.08 | 1.16 | 3.10 | 37.98 |  | 59.45 | 8.76 | 5.00 | 4.31 | 6.77 | 40.55 |  | 88.17 | 35.95 | 26.26 | 0.21 | 0.36 | 11.83 |
| 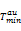 | 45.95 | 4.50 | 2.30 | 4.96 | 7.84 | 54.05 |  | 51.94 | 11.24 | 4.65 | 4.07 | 7.36 | 48.06 |  | 26.27 | 3.24 | 1.76 | 14.04 | 20.95 | 73.73 |  | 59.46 | 8.12 | 3.81 | 1.21 | 2.35 | 40.54 |
| 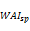 | 44.66 | 9.06 | 6.10 | 5.48 | 9.19 | 55.34 |  | 66.09 | 22.48 | 16.47 | 2.13 | 4.26 | 33.91 |  | 62.56 | 13.39 | 8.39 | 2.55 | 4.63 | 37.44 |  | 33.88 | 1.25 | 0.50 | 9.76 | 15.03 | 66.12 |
| 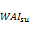 | 50.88 | 12.03 | 8.26 | 4.85 | 8.24 | 49.12 |  | 65.89 | 17.64 | 12.60 | 2.91 | 5.62 | 34.11 |  | 60.06 | 17.89 | 12.88 | 2.92 | 5.28 | 39.94 |  | 53.76 | 6.70 | 3.81 | 3.71 | 6.23 | 46.24 |
| 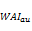 | 32.27 | 2.94 | 1.58 | 10.51 | 15.86 | 67.73 |  | 22.29 | 0.39 | 0.00 | 6.78 | 11.63 | 77.71 |  | 29.98 | 1.20 | 0.65 | 12.97 | 19.51 | 70.02 |  | 31.96 | 1.67 | 0.86 | 8.37 | 13.36 | 68.04 |

Note: %+ and %– indicate percentages of pixels showing positive and negative interannual sensitivity of total growing-season (April-October) GPP to seasonal climate. %10 and %5 indicate percentages of pixels showing significant responses of growing-season (April-October) GPP to seasonal climate at 0.1 and 0.05 level, respectively. *R* = ±0.39 and *R* = ±0.30 correspond to the 5% and 10% significance levels for the Spearman partial correlation between interannual variations of total growing-season GPP and seasonal mean climate, respectively. Seven major climate zones based on Köppen–Geiger climate classification are considered in this study: RegAR (arid region, grouping of BWk, BWh, BSk, BSh), RegTH (temperate humid region, grouping of Cfa, Cfb, and Cfc), RegTA (temperate dry region, grouping of Csa, Csb, Csc, Cwa, Cwb and Cwc), RegAH (cold humid region, grouping of Dfa, Dfb, Dfc, and Dfd), RegAS (cold summer dry region, grouping of Dsa, Dsb, Dsc, and, Dsd), RegAW (cold winter dry region, grouping of Dwa, Dwb, Dwc, and, Dwd), and RegET (polar tundra, ET).

**Table S4**. Geographic locations and chronology characteristics for tree ring increment observations.

| Serie No | SiteID | Geography Location | | |  | Chronology Characteristics | | | |  | Data Source |
| --- | --- | --- | --- | --- | --- | --- | --- | --- | --- | --- | --- |
|  |  | Latitude | Longitude | Altitude (m) |  | Start year | End year | Species^*^ | No. Cores |  | Data Source^ǂ^ |
| 1 | JAPA011 | 44.02 | 143.83 | 200 |  | 1693 | 2003 | PCGN | 58 |  | ITRDB |
| 2 | KORE001 | 38.13 | 128.47 | 1500 |  | 1657 | 1998 | PIKO | 86 |  | ITRDB |
| 3 | LEBA001 | 33.68 | 35.68 | 1775 |  | 1829 | 2002 | CDLI | 31 |  | ITRDB |
| 4 | LEBA002 | 34.47 | 36.23 | 1175 |  | 1722 | 2001 | ABCI | 18 |  | ITRDB |
| 5 | LEBA003 | 34.30 | 35.98 | 1640 |  | 1809 | 2001 | CDLI | 22 |  | ITRDB |
| 6 | LEBA004 | 34.23 | 36.03 | 1900 |  | 1382 | 2002 | CDLI | 55 |  | ITRDB |
| 7 | LEBA005 | 34.13 | 35.82 | 1780 |  | 1778 | 2002 | CDLI | 52 |  | ITRDB |
| 8 | LEBA006 | 33.67 | 35.68 | 1720 |  | 1730 | 2002 | CDLI | 18 |  | ITRDB |
| 9 | MONG011 | 48.15 | 100.28 | 1900 |  | 1513 | 2001 | LASI | 36 |  | ITRDB |
| 10 | MONG012 | 48.98 | 103.23 | 1400 |  | 1511 | 2002 | LASI | 25 |  | ITRDB |
| 11 | MONG013 | 48.77 | 97.12 | 1841 |  | 1638 | 1998 | LASI | 28 |  | ITRDB |
| 12 | MONG014 | 49.48 | 100.83 | 1800 |  | 1557 | 2002 | LASI | 27 |  | ITRDB |
| 13 | RUSS219 | 43.25 | 145.98 | 367 |  | 1585 | 2000 | QUMO | 31 |  | ITRDB |
| 14 | SYRI001 | 35.60 | 36.22 | 1450 |  | 1837 | 2001 | CDLI | 20 |  | ITRDB |
| 15 | SYRI002 | 35.57 | 36.20 | 1450 |  | 1795 | 2001 | ABCI | 35 |  | ITRDB |
| 16 | SYRI003 | 35.78 | 36.02 | 480 |  | 1882 | 2001 | PIBR | 40 |  | ITRDB |
| 17 | CANA150 | 49.87 | -118.85 | 1700 |  | 1689 | 1998 | PCEN | 46 |  | ITRDB |
| 18 | CANA151 | 49.87 | -118.85 | 1700 |  | 1669 | 1998 | PICO | 23 |  | ITRDB |
| 19 | CANA152 | 49.87 | -118.85 | 1700 |  | 1725 | 1998 | ABLA | 50 |  | ITRDB |
| 20 | CANA174 | 50.22 | -126.35 | 1005 |  | 1394 | 1999 | ABAM | 64 |  | ITRDB |
| 21 | CANA175 | 50.22 | -126.35 | 1005 |  | 1200 | 1999 | CHNO | 66 |  | ITRDB |
| 22 | CANA195 | 49.83 | -97.20 | 230 |  | 1286 | 1999 | QUMA | 354 |  | ITRDB |
| 23 | CANA211 | 61.90 | -140.72 | 731 |  | 1679 | 1999 | PCGL | 17 |  | ITRDB |
| 24 | CANA269 | 46.83 | -71.17 | 30 |  | 1540 | 2005 | THOC | 21 |  | ITRDB |
| 25 | CANA283 | 62.57 | -114.35 | 209 |  | 1873 | 2005 | PIBN | 44 |  | ITRDB |
| 26 | CANA284 | 62.52 | -114.35 | 189 |  | 1862 | 2005 | PIBN | 54 |  | ITRDB |
| 27 | CANA285 | 62.50 | -114.35 | 194 |  | 1864 | 2005 | PIBN | 44 |  | ITRDB |
| 28 | CANA286 | 62.48 | -114.37 | 211 |  | 1858 | 2005 | PIBN | 46 |  | ITRDB |
| 29 | CANA287 | 62.48 | -114.42 | 208 |  | 1679 | 2005 | PIBN | 40 |  | ITRDB |
| 30 | CANA288 | 62.42 | -114.42 | 175 |  | 1828 | 2005 | PIBN | 44 |  | ITRDB |
| 31 | CANA289 | 62.47 | -114.30 | 191 |  | 1806 | 2005 | PIBN | 30 |  | ITRDB |
| 32 | CANA290 | 62.60 | -114.13 | 195 |  | 1829 | 2005 | PIBN | 52 |  | ITRDB |
| 33 | CANA291 | 62.55 | -113.85 | 197 |  | 1853 | 2005 | PIBN | 46 |  | ITRDB |
| 34 | CANA292 | 62.52 | -113.82 | 199 |  | 1936 | 2005 | PIBN | 39 |  | ITRDB |
| 35 | CANA293 | 62.55 | -113.87 | 214 |  | 1734 | 2005 | PIBN | 51 |  | ITRDB |
| 36 | CANA294 | 62.50 | -113.43 | 228 |  | 1804 | 2005 | PIBN | 48 |  | ITRDB |
| 37 | BRIT053 | 53.37 | -1.50 | 140 |  | 1759 | 2003 | QURO | 20 |  | ITRDB |
| 38 | BRIT054 | 53.35 | -6.32 | 52 |  | 1666 | 2008 | QUSP | 14 |  | ITRDB |
| 39 | CYPR015 | 35.02 | 32.63 | 1050 |  | 1739 | 2002 | PIBR | 37 |  | ITRDB |
| 40 | CYPR016 | 34.92 | 32.90 | 1550 |  | 1584 | 2002 | PIBR | 30 |  | ITRDB |
| 41 | CYPR017 | 34.92 | 32.90 | 1640 |  | 1554 | 2002 | PINI | 23 |  | ITRDB |
| 42 | CYPR018 | 34.93 | 32.87 | 1770 |  | 1379 | 2002 | PINI | 34 |  | ITRDB |
| 43 | CYPR019 | 34.98 | 32.67 | 1400 |  | 1532 | 2002 | CDBR | 41 |  | ITRDB |
| 44 | GERM034 | 48.92 | 12.58 | 370 |  | 1837 | 1998 | PCAB | 45 |  | ITRDB |
| 45 | GREE008 | 40.30 | 20.90 | 1500 |  | 1751 | 2003 | PINI | 26 |  | ITRDB |
| 46 | GREE009 | 36.92 | 22.35 | 1400 |  | 1657 | 1999 | PINI | 29 |  | ITRDB |
| 47 | LITH011 | 55.07 | 22.48 | 25 |  | 1878 | 2002 | QURO | 20 |  | ITRDB |
| 48 | LITH012 | 55.97 | 21.08 | 12 |  | 1816 | 2002 | PISY | 39 |  | ITRDB |
| 49 | LITH013 | 55.43 | 26.03 | 140 |  | 1890 | 2003 | PISY | 93 |  | ITRDB |
| 50 | SPAI054 | 39.38 | -2.63 | 700 |  | 1924 | 1999 | PIPN | 25 |  | ITRDB |
| 51 | SPAI055 | 39.38 | -2.63 | 700 |  | 1920 | 1999 | PIPN | 22 |  | ITRDB |
| 52 | SPAI056 | 39.33 | -2.42 | 720 |  | 1882 | 1999 | PIPN | 13 |  | ITRDB |
| 53 | SPAI057 | 40.67 | -2.77 | 1055 |  | 1874 | 2001 | PIPN | 22 |  | ITRDB |
| 54 | SPAI059 | 39.28 | -1.35 | 705 |  | 1907 | 2001 | PIPN | 31 |  | ITRDB |
| 55 | SWED317 | 64.53 | 19.00 | 290 |  | 1724 | 1998 | ISY | 12 |  | ITRDB |
| 56 | SWED319 | 67.63 | 21.77 | 280 |  | 1656 | 1998 | ISY | 21 |  | ITRDB |
| 57 | SWED324 | 62.33 | 18.48 | 250 |  | 1448 | 1998 | PISY | 36 |  | ITRDB |
| 58 | SWED325 | 63.98 | 16.53 | 270 |  | 1520 | 1999 | ISY | 33 |  | ITRDB |
| 59 | SWED326 | 63.12 | 13.33 | 700 |  | 1471 | 1998 | PISY | 36 |  | ITRDB |
| 60 | SWED327 | 64.45 | 13.97 | 470 |  | 1500 | 2000 | PISY | 19 |  | ITRDB |
| 61 | SWED328 | 59.18 | 18.27 | 60 |  | 1694 | 2000 | PISY | 18 |  | ITRDB |
| 62 | TURK001 | 40.00 | 31.08 | 1400 |  | 1292 | 2001 | PINI | 36 |  | ITRDB |
| 63 | TURK010 | 39.80 | 27.13 | 1200 |  | 1556 | 1999 | PINI | 16 |  | ITRDB |
| 64 | TURK011 | 37.23 | 28.38 | 1200 |  | 1568 | 1999 | PINI | 14 |  | ITRDB |
| 65 | TURK013 | 37.42 | 30.28 | 1601 |  | 1511 | 2001 | PINI | 35 |  | ITRDB |
| 66 | TURK014 | 37.42 | 30.30 | 1862 |  | 1246 | 2000 | JUEX | 17 |  | ITRDB |
| 67 | TURK015 | 37.40 | 30.63 | 1156 |  | 1730 | 2000 | IBR | 30 |  | ITRDB |
| 68 | TURK016 | 36.60 | 30.02 | 1853 |  | 1017 | 2006 | JUEX | 81 |  | ITRDB |
| 69 | TURK017 | 36.60 | 30.02 | 1937 |  | 1449 | 2000 | CDLI | 35 |  | ITRDB |
| 70 | TURK018 | 37.08 | 30.52 | 1047 |  | 1152 | 2000 | UEX | 27 |  | ITRDB |
| 71 | TURK019 | 37.38 | 30.60 | 1469 |  | 1693 | 2000 | CDLI | 25 |  | ITRDB |
| 72 | TURK020 | 36.65 | 32.20 | 1633 |  | 1586 | 2000 | PINI | 24 |  | ITRDB |
| 73 | TURK021 | 36.65 | 32.18 | 1723 |  | 1628 | 2000 | CDLI | 24 |  | ITRDB |
| 74 | TURK023 | 36.45 | 32.52 | 1580 |  | 1444 | 2003 | PINI | 21 |  | ITRDB |
| 75 | TURK025 | 36.45 | 32.52 | 1770 |  | 1423 | 2003 | CDLI | 23 |  | ITRDB |
| 76 | TURK028 | 41.20 | 32.62 | 900 |  | 1699 | 2004 | QUPE | 19 |  | ITRDB |
| 77 | TURK030 | 39.28 | 28.93 | 1600 |  | 1771 | 2002 | PINI | 79 |  | ITRDB |
| 78 | TURK031 | 37.63 | 35.43 | 1500 |  | 1475 | 2001 | PINI | 18 |  | ITRDB |
| 79 | TURK032 | 37.03 | 30.47 | 700 |  | 1738 | 2001 | PIBR | 15 |  | ITRDB |
| 80 | TURK036 | 37.15 | 30.52 | 1047 |  | 1694 | 2000 | IBR | 35 |  | ITRDB |
| 81 | AKST075 | 61.15 | -142.08 | 1030 |  | 1580 | 1998 | PCGL | 27 |  | ITRDB |
| 82 | AKST086 | 60.48 | -154.33 | 550 |  | 1627 | 2003 | PCGL | 36 |  | ITRDB |
| 83 | AKST087 | 60.50 | -153.88 | 580 |  | 1672 | 2003 | PCGL | 51 |  | ITRDB |
| 84 | AKST088 | 60.97 | -153.92 | 400 |  | 1769 | 2003 | PCGL | 20 |  | ITRDB |
| 85 | AKST089 | 60.65 | -154.02 | 580 |  | 1600 | 2003 | PCGL | 33 |  | ITRDB |
| 86 | AKST091 | 61.05 | -147.10 | 90 |  | 1406 | 2002 | TSME | 73 |  | ITRDB |
| 87 | AKST093 | 61.05 | -146.98 | 200 |  | 1472 | 1999 | TSME | 33 |  | ITRDB |
| 88 | AKST096 | 61.05 | -146.98 | 200 |  | 616 | 2002 | TSME | 297 |  | ITRDB |
| 89 | CAST614 | 35.30 | -120.27 | 561 |  | 1455 | 2004 | QUDG | 91 |  | ITRDB |
| 90 | CAST615 | 39.02 | -122.82 | 426 |  | 1620 | 2004 | QUDG | 99 |  | ITRDB |
| 91 | CAST616 | 37.72 | -120.42 | 274 |  | 1531 | 2005 | QUDG | 64 |  | ITRDB |
| 92 | CAST618 | 39.52 | -121.43 | 305 |  | 1572 | 2004 | QUDG | 99 |  | ITRDB |
| 93 | CAST619 | 40.27 | -121.85 | 853 |  | 1393 | 2004 | QUDG | 103 |  | ITRDB |
| 94 | CAST620 | 38.77 | -121.10 | 167 |  | 1603 | 2003 | QUDG | 117 |  | ITRDB |
| 95 | CAST621 | 35.52 | -118.67 | 670 |  | 1585 | 2003 | QUDG | 75 |  | ITRDB |
| 96 | CAST623 | 37.87 | -121.95 | 185 |  | 1582 | 2004 | QUDG | 82 |  | ITRDB |
| 97 | CAST625 | 37.05 | -121.35 | 396 |  | 1510 | 2003 | QUDG | 109 |  | ITRDB |
| 98 | CAST626 | 36.47 | -121.18 | 350 |  | 1577 | 2003 | QUDG | 82 |  | ITRDB |
| 99 | CAST628 | 40.15 | -120.60 | 1480 |  | 1450 | 1998 | PIPO | 30 |  | ITRDB |
| 100 | CAST629 | 41.45 | -120.90 | 1302 |  | 1152 | 1998 | JUOC | 14 |  | ITRDB |
| 101 | CAST631 | 39.52 | -120.55 | 1921 |  | 930 | 1999 | JUOC | 12 |  | ITRDB |
| 102 | CAST634 | 37.63 | -121.77 | 149 |  | 1816 | 2003 | PLRA | 32 |  | ITRDB |
| 103 | CAST635 | 37.63 | -121.77 | 149 |  | 1896 | 2003 | QULO | 22 |  | ITRDB |
| 104 | CAST645 | 37.67 | -122.30 | 475 |  | 1697 | 2003 | QUDG | 42 |  | ITRDB |
| 105 | CAST646 | 36.82 | -121.47 | 1067 |  | 1379 | 2003 | QUDG | 48 |  | ITRDB |
| 106 | CAST647 | 36.57 | -120.87 | 1219 |  | 1409 | 2003 | QUDG | 28 |  | ITRDB |
| 107 | CAST648 | 40.88 | -120.03 | 396 |  | 1582 | 2004 | QUDG | 44 |  | ITRDB |
| 108 | CAST649 | 39.35 | -122.73 | 468 |  | 1546 | 2004 | QUDG | 42 |  | ITRDB |
| 109 | CAST650 | 40.47 | -122.75 | 421 |  | 1649 | 2004 | QUDG | 14 |  | ITRDB |
| 110 | CAST651 | 36.48 | -119.30 | 1132 |  | 1596 | 2003 | QUDG | 51 |  | ITRDB |
| 111 | CAST652 | 40.42 | -122.63 | 218 |  | 1519 | 2004 | QUDG | 42 |  | ITRDB |
| 112 | CAST653 | 36.52 | -119.12 | 1237 |  | 1448 | 2003 | QUDG | 62 |  | ITRDB |
| 113 | CAST654 | 40.15 | -122.05 | 143 |  | 1601 | 2005 | QUDG | 55 |  | ITRDB |
| 114 | CAST655 | 38.00 | -121.02 | 457 |  | 1695 | 2004 | QUDG | 30 |  | ITRDB |
| 115 | CAST656 | 35.23 | -120.00 | 1036 |  | 1293 | 2003 | QUDG | 55 |  | ITRDB |
| 116 | CAST657 | 36.65 | -121.92 | 755 |  | 1460 | 2004 | QUDG | 60 |  | ITRDB |
| 117 | CAST658 | 36.20 | -121.77 | 646 |  | 1494 | 2003 | QUDG | 34 |  | ITRDB |
| 118 | CAST659 | 36.92 | -119.48 | 701 |  | 1494 | 2004 | QUDG | 41 |  | ITRDB |
| 119 | CAST660 | 35.53 | -119.40 | 1051 |  | 1333 | 2004 | QUDG | 34 |  | ITRDB |
| 120 | CAST661 | 40.40 | -123.02 | 342 |  | 1532 | 2004 | QUDG | 51 |  | ITRDB |
| 121 | CAST662 | 36.15 | -120.43 | 558 |  | 1538 | 2004 | QUDG | 29 |  | ITRDB |
| 122 | CAST663 | 39.12 | -122.45 | 180 |  | 1534 | 2004 | QUDG | 21 |  | ITRDB |
| 123 | CAST664 | 37.15 | -120.25 | 345 |  | 1557 | 2004 | QUDG | 54 |  | ITRDB |
| 124 | CAST665 | 38.45 | -120.63 | 1194 |  | 1408 | 2004 | QUDG | 37 |  | ITRDB |
| 125 | COST582 | 38.67 | -108.35 | 1737 |  | 1569 | 1999 | PIED | 28 |  | ITRDB |
| 126 | COST600 | 38.25 | -108.33 | 1996 |  | 1536 | 2000 | PIED | 32 |  | ITRDB |
| 127 | COST608 | 39.68 | -105.20 | 1965 |  | 1487 | 2003 | PIPO | 22 |  | ITRDB |
| 128 | COST640 | 38.40 | -105.30 | 1900 |  | 1577 | 2003 | PIED | 24 |  | ITRDB |
| 129 | COST641 | 39.80 | -105.25 | 1920 |  | 1566 | 2003 | PIPO | 19 |  | ITRDB |
| 130 | NEST005 | 42.70 | -100.87 | 810 |  | 1728 | 1998 | PIPO | 35 |  | ITRDB |
| 131 | NHST005 | 43.80 | -71.83 | 300 |  | 1690 | 2008 | PIRE | 47 |  | ITRDB |
| 132 | ORST085 | 45.50 | -121.42 | 1002 |  | 1602 | 1999 | PIPO | 43 |  | ITRDB |
| 133 | SDST020 | 45.28 | -100.73 | 501 |  | 1840 | 2006 | PPDE | 25 |  | ITRDB |
| 134 | TXST051 | 33.40 | -98.08 | 275 |  | 1793 | 2006 | QUST | 51 |  | ITRDB |
| 135 | VAST027 | 37.55 | -79.07 | 230 |  | 1749 | 1998 | QUAL | 17 |  | ITRDB |
| 136 | WIST006 | 42.67 | -87.90 | 217 |  | 1807 | 2000 | QUAL | 15 |  | ITRDB |
| 137 | CHIN001 | 43.82 | 93.31 | 1834 |  | 1924 | 2000 | LASI | 65 |  | This Study |
| 138 | CHIN002 | 43.82 | 93.34 | 1954 |  | 1897 | 2000 | LASI | 63 |  | This Study |
| 139 | CHIN003 | 43.89 | 88.67 | 1886 |  | 1915 | 2005 | PCSH | 119 |  | This Study |
| 140 | CHIN004 | 43.90 | 88.68 | 1995 |  | 1902 | 2005 | PCSH | 49 |  | This Study |
| 141 | CHIN005 | 43.28 | 87.18 | 1728 |  | 1923 | 2005 | PCSH | 42 |  | This Study |
| 142 | CHIN006 | 43.43 | 87.24 | 1819 |  | 1938 | 2005 | PCSH | 46 |  | This Study |
| 143 | CHIN007 | 43.24 | 116.39 | 1363 |  | 1876 | 2004 | PITB | 42 |  | Liang *et al*. 2007[^1^](#_ENREF_8_1) |
| 144 | CHIN008 | 43.09 | 116.60 | 1331 |  | 1840 | 2004 | PITB | 29 |  | This Study |
| 145 | CHIN009 | 43.90 | 116.96 | 1285 |  | 1920 | 2004 | PITB | 40 |  | This Study |
| 146 | CHIN010 | 42.90 | 116.96 | 1250 |  | 1930 | 2004 | PITB | 40 |  | This Study |
| 147 | CHIN011 | 42.99 | 117.05 | 1329 |  | 1868 | 2004 | PITB | 41 |  | This Study |
| 148 | CHIN012 | 43.63 | 117.72 | 1197 |  | 1873 | 2004 | PITB | 40 |  | This Study |
| 149 | CHIN013 | 39.50 | 110.67 | 1354 |  | 1912 | 2003 | PITB | 41 |  | This Study |

* The species codes are coincide with that of The International Tree-Ring Data Bank (ITRDB).

ǂ ITRDB (available from: http://www.ncdc.noaa.gov/data-access/paleoclimatology-data/datasets/tree-ring)

Figure S1


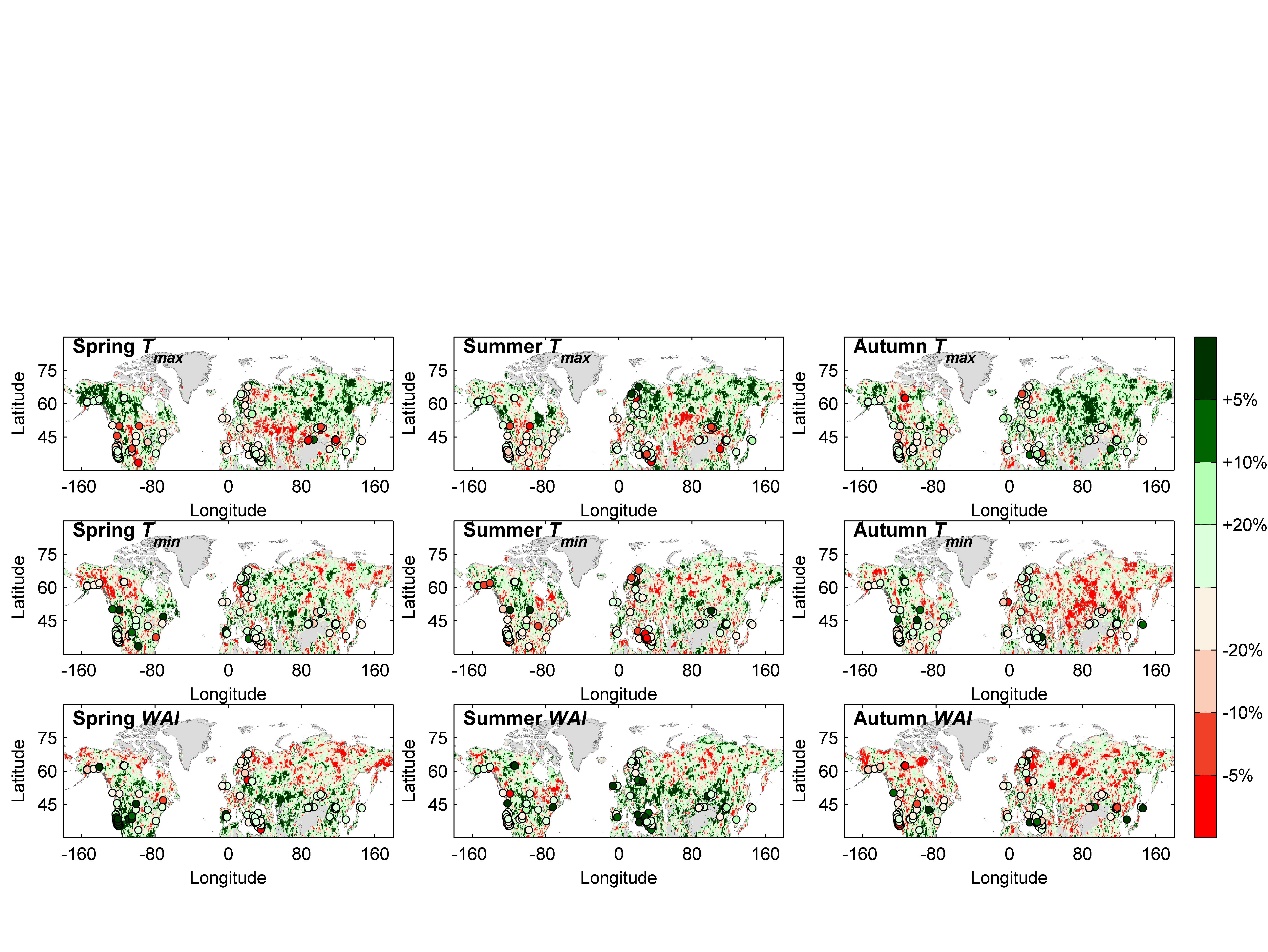


**Figure S1. Spatial patterns of the interannual responses of the mean growing-season (April-October) NDVI and TRI to seasonal VDNC in the mid- and high-latitude NH.** Spearman partial correlation coefficients between the mean growing-season NDVI (
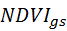
) (during 1982-2008) and TRI (dots) (during 1950-2008, if available) as well as between the seasonal mean maximum temperature (
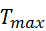
), mean minimum temperature (
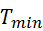
) and water availability index (WAI) from spring to autumn are shown. The labels 5%, 10% and 20% in the color bar indicate the corresponding significance level of Student’s t-test for the partial correlation analyses between interannual variations of
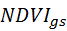
 and TRI and seasonal mean climate. R = ±0.39, R = ±0.30, and R = ±0.20 correspond to the 5%, 10% and 20% significance levels for the Spearman partial correlation between interannual variations of
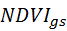
 and seasonal mean climate, respectively. This figure is created by MATLAB (R2012b).

**Figure S2**


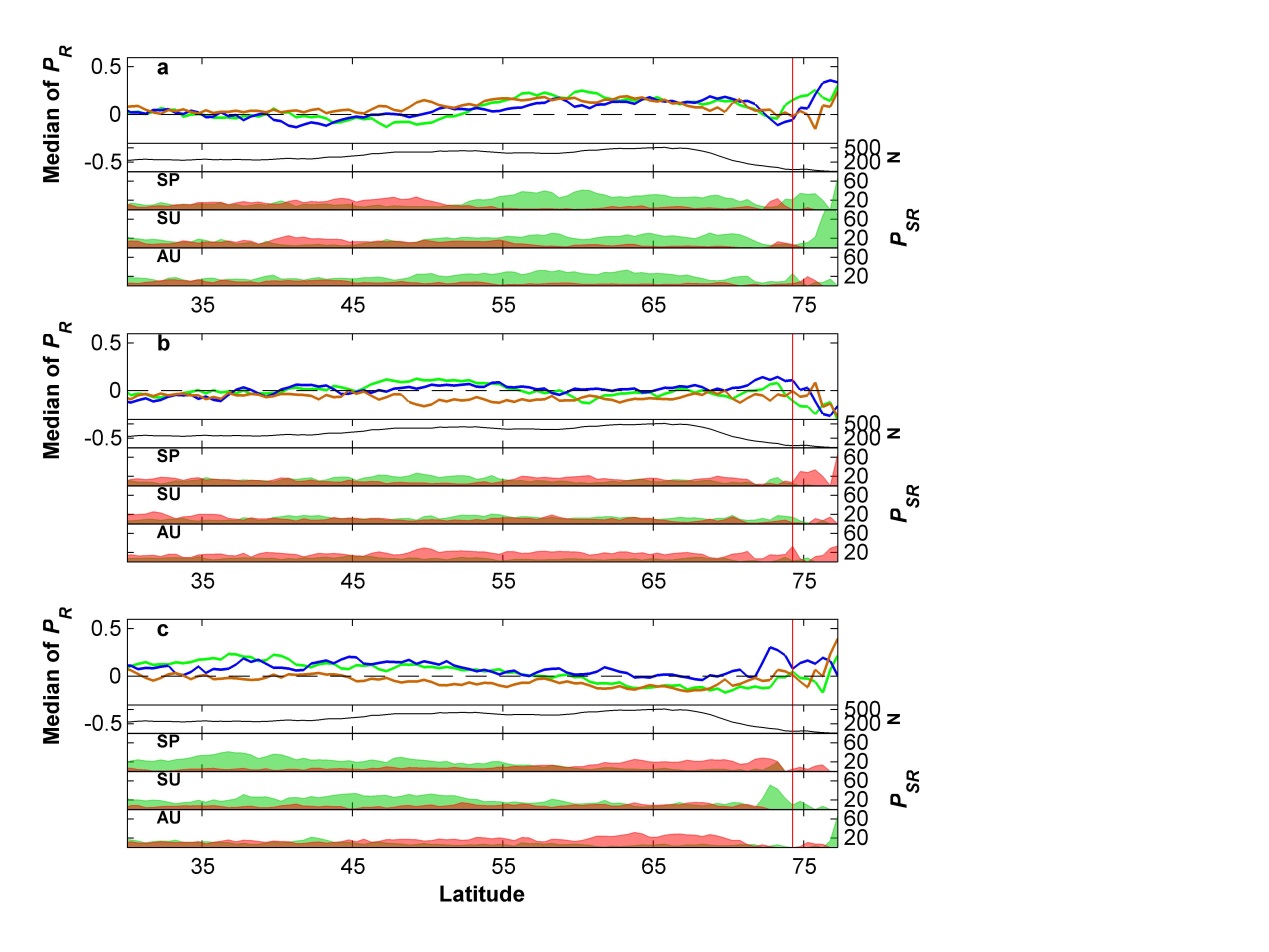


**Figure S2. Latitudinal patterns of the interannual responses of growing season (April-October) NDVI to IAV of seasonal variations of maximum and minimum temperature and water availability index.** Latitudinal patterns of the median of partial correlation coefficients (*P_R_*) between
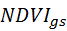
and spring (green lines), summer (blue lines), and autumn (brown lines) maximum temperature (a), minimum temperature (b) and water availability index (c). Percentage of pixels (within every 0.5 degree interval) showing significant (*p* < 0.1) positive and negative correlation (*P_SR_*) of
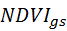
 to spring (SP), summer (SU) and autumn (AU) maximum temperature (a), minimum temperature (b) and water availability index (c) are indicated by the filled green and red areas, respectively. The black lines in inlets of a, b and c show the sampling depths (N) within each 0.5 degree interval. The vertical red lines in a), b) and c) indicate the latitudes north than it the sample depths are lower than 50.

**Figure S3**


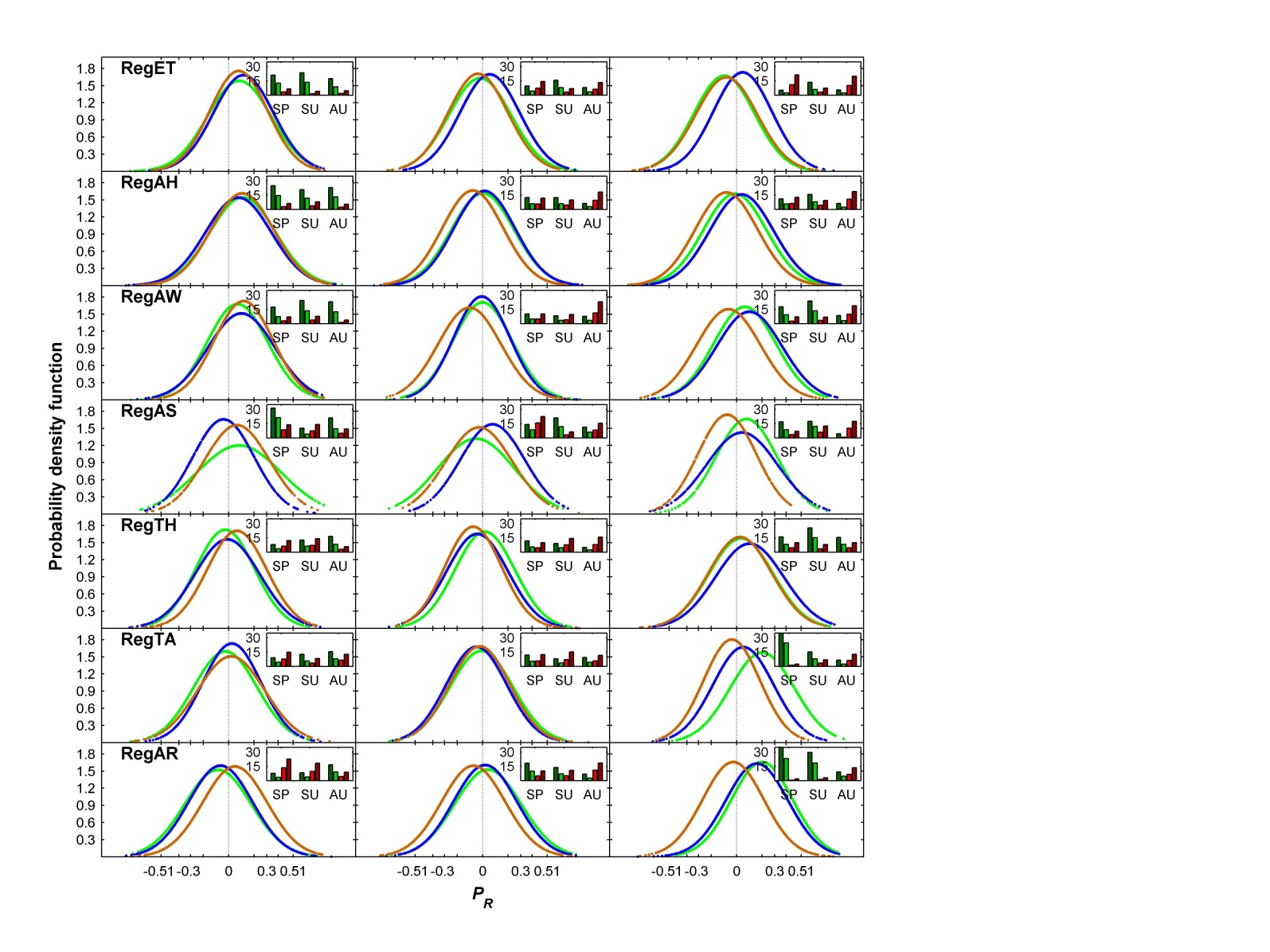


**Figure S3.** **Probability density functions of partial correlation coefficients between growing-season NDVI and seasonal variations of maximum and minimum temperature and water availability index in different climate regions.** Probability density functions (PDF) of partial correlation coefficients (*P_R_*) between growing-season NDVI and spring (green lines), summer (blue lines) and autumn (brown lines) maximum temperature (left column), minimum temperature (middle column) and water availability index (right column). Percentage of pixels showing significant positive (dark and light green bars correspond to 10% and 5% significant level, respectively) and negative (dark and light red bars correspond to 10% and 5% significant level, respectively) correlation of growing-season NDVI to spring (SP), summer (SU) and autumn (AU) maximum temperature, minimum temperature and water availability index are shown in inlets, respectively. The Spearman correlation coefficients *R* ±0.51 and ±0.30 in *x* axis tick label correspond to 1% and 10% significance level of student’s t-test, respectively.

**Figure S4**.


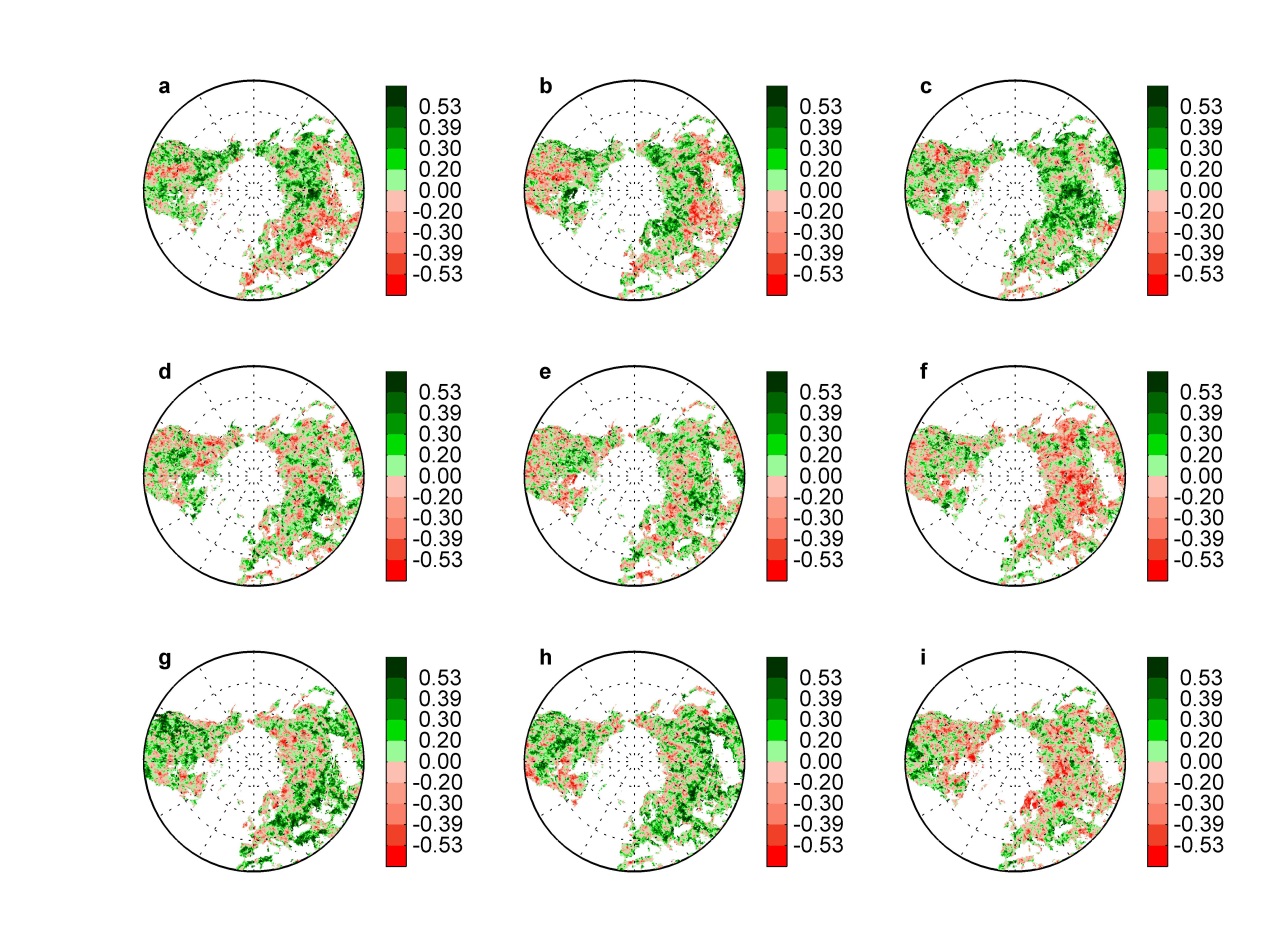


**Figure S4. Spatial patterns of the responses of growing-season (April-October) FAPAR to IAV of seasonal variations of maximum and minimum temperature and water availability index in mid- and high-latitudinal Northern Hemisphere during 1982-2008.** Spearman partial correlation coefficients between growing-season FAPAR, *FAPAR_gs_*, and
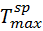
 (spring maximum temperature),
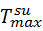
 (summer maximum temperature),
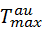
 (autumn maximum temperature),
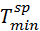
 (spring minimum temperature),
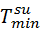
 (summer minimum temperature),
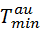
 (autumn minimum temperature) are shown in a-f, respectively. Spearman partial correlation coefficients between *FAPAR_gs_* and spring, summer and autumn *WAI* are shown in g-i, respectively. The Spearman correlation coefficients *R* ±0.53, ±0.39, ±0.30, and ±0.20 correspond to 1%, 5%, 10% and 20% significance level of student’s t-test, respectively. Blank regions are masked from our analysis (in detail see Methods). The map in this figure is created by MATLAB (R2012b).

**Figure S5**.

**
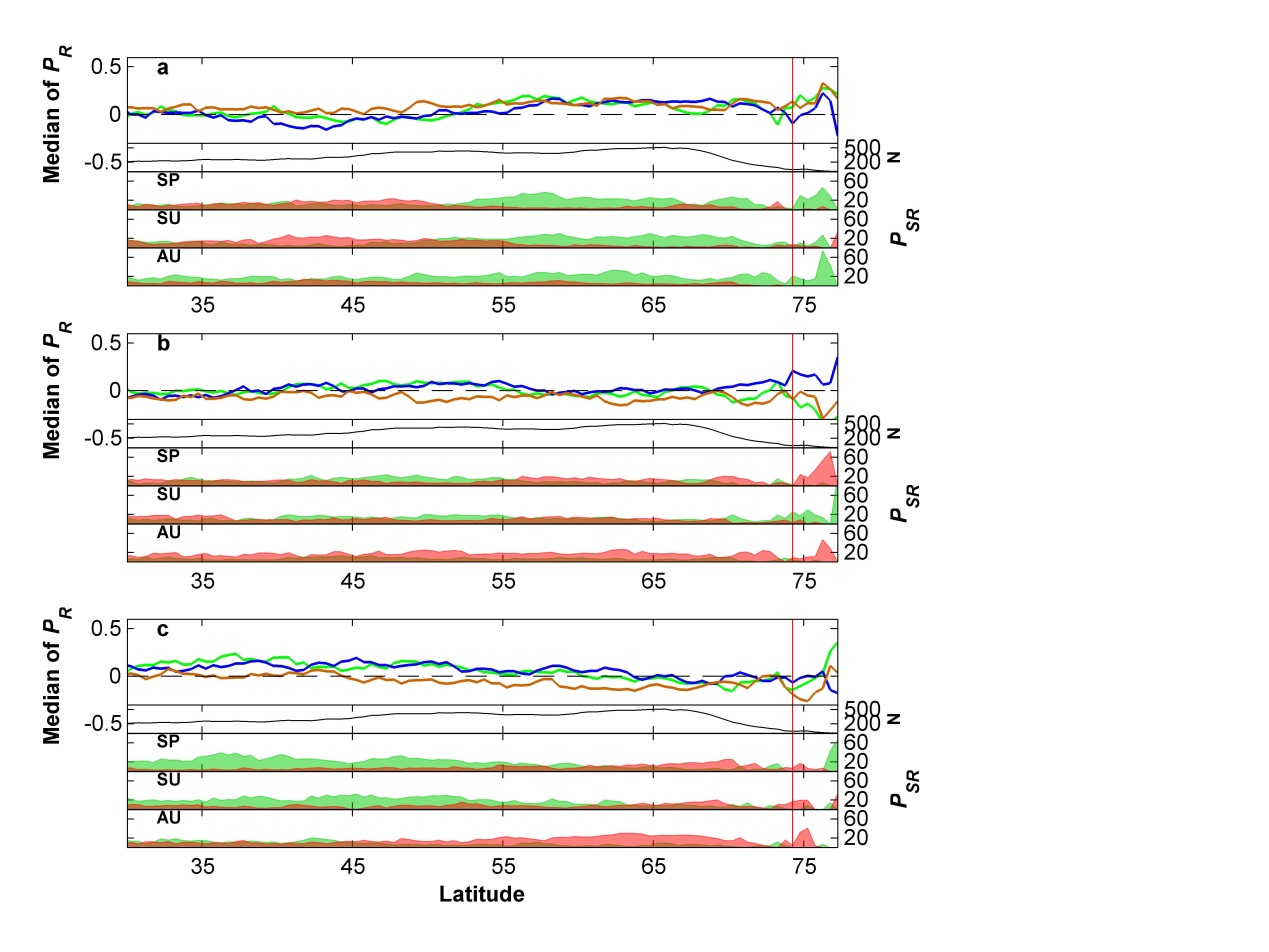
**

**Figure S5. Latitudinal patterns of the responses of growing season (April-October) FAPAR to IAV of seasonal variations of maximum and minimum temperature and water availability index.** Latitudinal patterns of the median of partial correlation coefficients (*P_R_*) between *FAPAR_gs_* and spring (green lines), summer (blue lines), and autumn (brown lines) maximum temperature (a), minimum temperature (b) and water availability index (c). Percentage of pixels (within every 0.5 degree interval) showing significant (*p* < 0.1) positive and negative correlation (*P_SR_*) of *FAPAR_gs_* to spring (SP), summer (SU) and autumn (AU) maximum temperature (a), minimum temperature (b) and water availability index (c) are indicated by the filled green and red areas, respectively. The black lines in inlets of a, b and c show the sampling depths (N) within each 0.5 degree interval. The vertical red lines in a), b) and c) indicate the latitudes north than it the sample depths are lower than 50.

**Figure S6.**


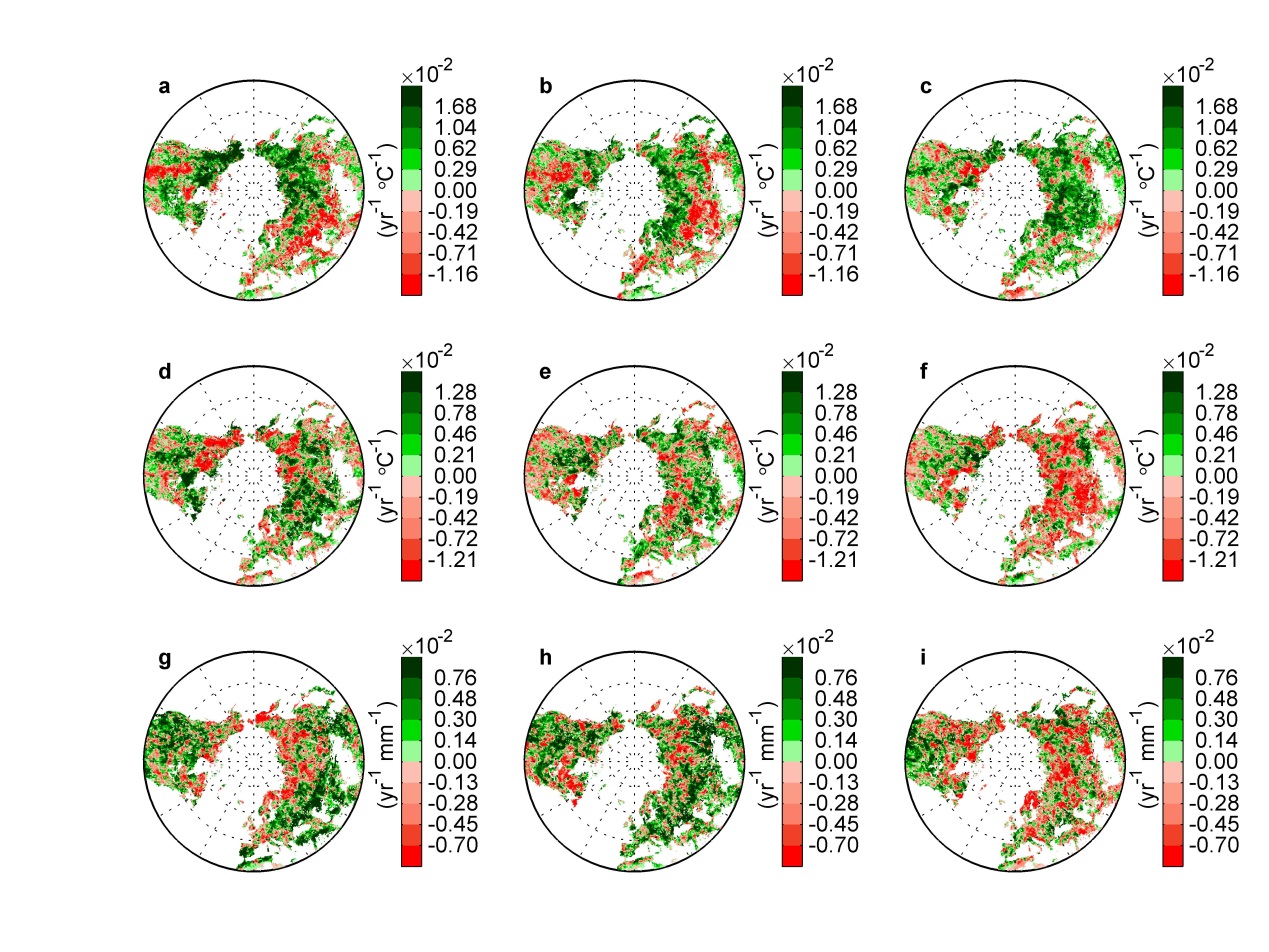


**Figure S6.** **Spatial patterns of the interannual sensitivity of growing-season (April-October) NDVI to seasonal variations of maximum and minimum temperature and water availability index in mid- and high-latitudinal Northern Hemisphere during 1982-2008.** Interannual sensitivity of
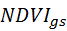
 to IAV of seasonal climate is estimated by ridge regression (in detail see Methods). Interannual sensitivity of
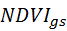
 to
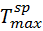
 (spring maximum temperature),
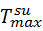
 (summer maximum temperature),
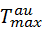
 (autumn maximum temperature),
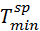
 (spring minimum temperature),
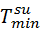
 (summer minimum temperature), and
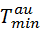
 (autumn minimum temperature) are shown in a-f, respectively. Sensitivity of
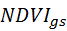
 to spring, summer and autumn *WAI* are shown in g-i, respectively. Blank regions are masked from our analysis (in detail see Methods). The map in this figure is created by MATLAB (R2012b).

**Figure S7**


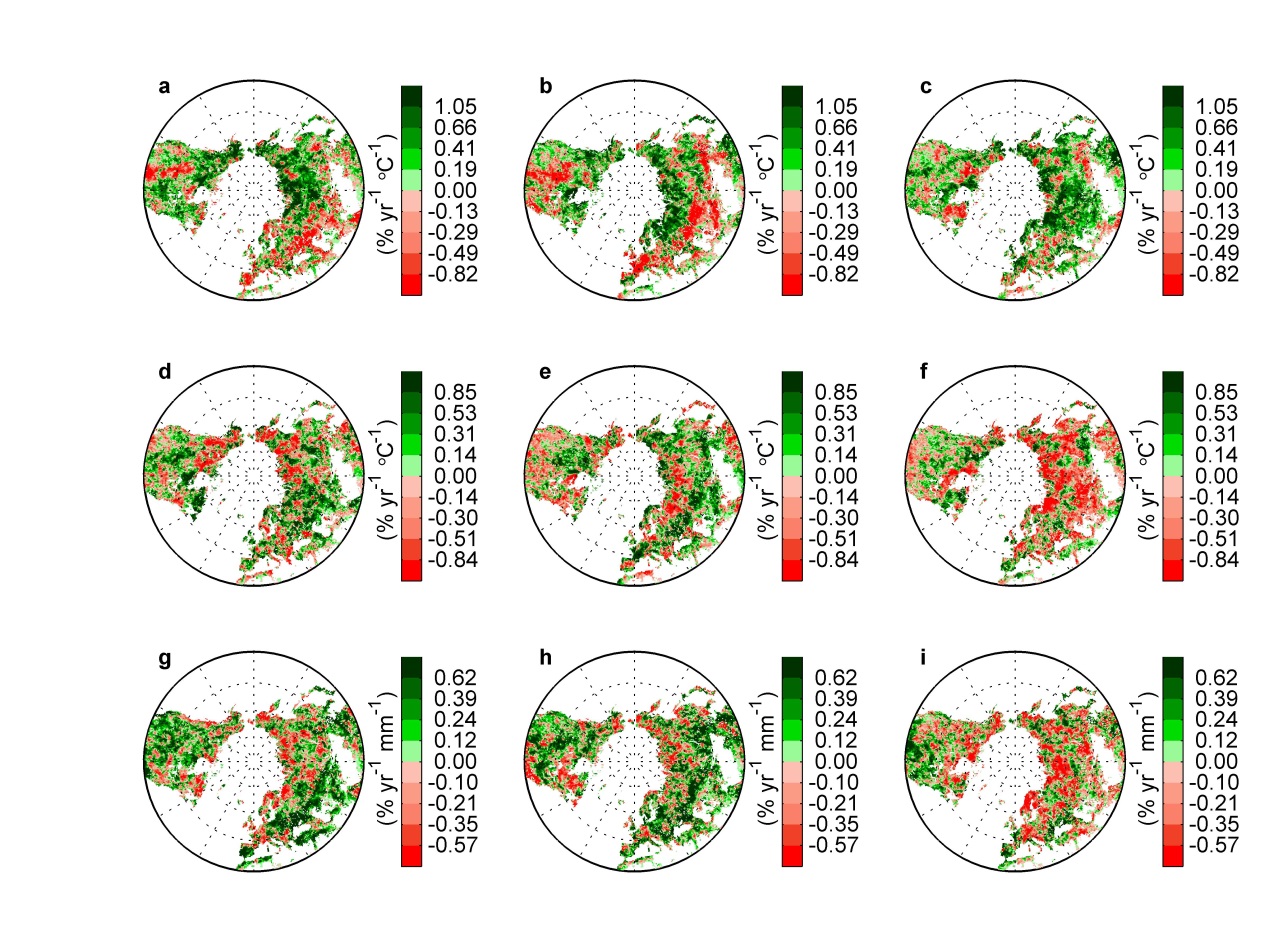


**Figure S7.** **Spatial patterns of the interannual sensitivity of growing-season (April-October) FAPAR to seasonal variations of maximum and minimum temperature and water availability index in mid- and high-latitudinal Northern Hemisphere during 1982-2008.** Interannual sensitivity of
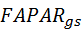
 (with unit of %) to IAV of seasonal climate is estimated by ridge regression (in detail see Methods). Interannual sensitivity of
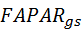
 to
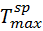
 (spring maximum temperature),
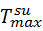
 (summer maximum temperature),
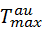
 (autumn maximum temperature),
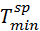
 (spring minimum temperature),
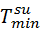
 (summer minimum temperature), and
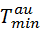
 (autumn minimum temperature) are shown in a-f, respectively. Sensitivity of
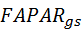
 to spring, summer and autumn *WAI* are shown in g-i, respectively. Blank regions are masked from our analysis (in detail see materials and methods). The map in this figure is created by MATLAB (R2012b).

**Figure S8**


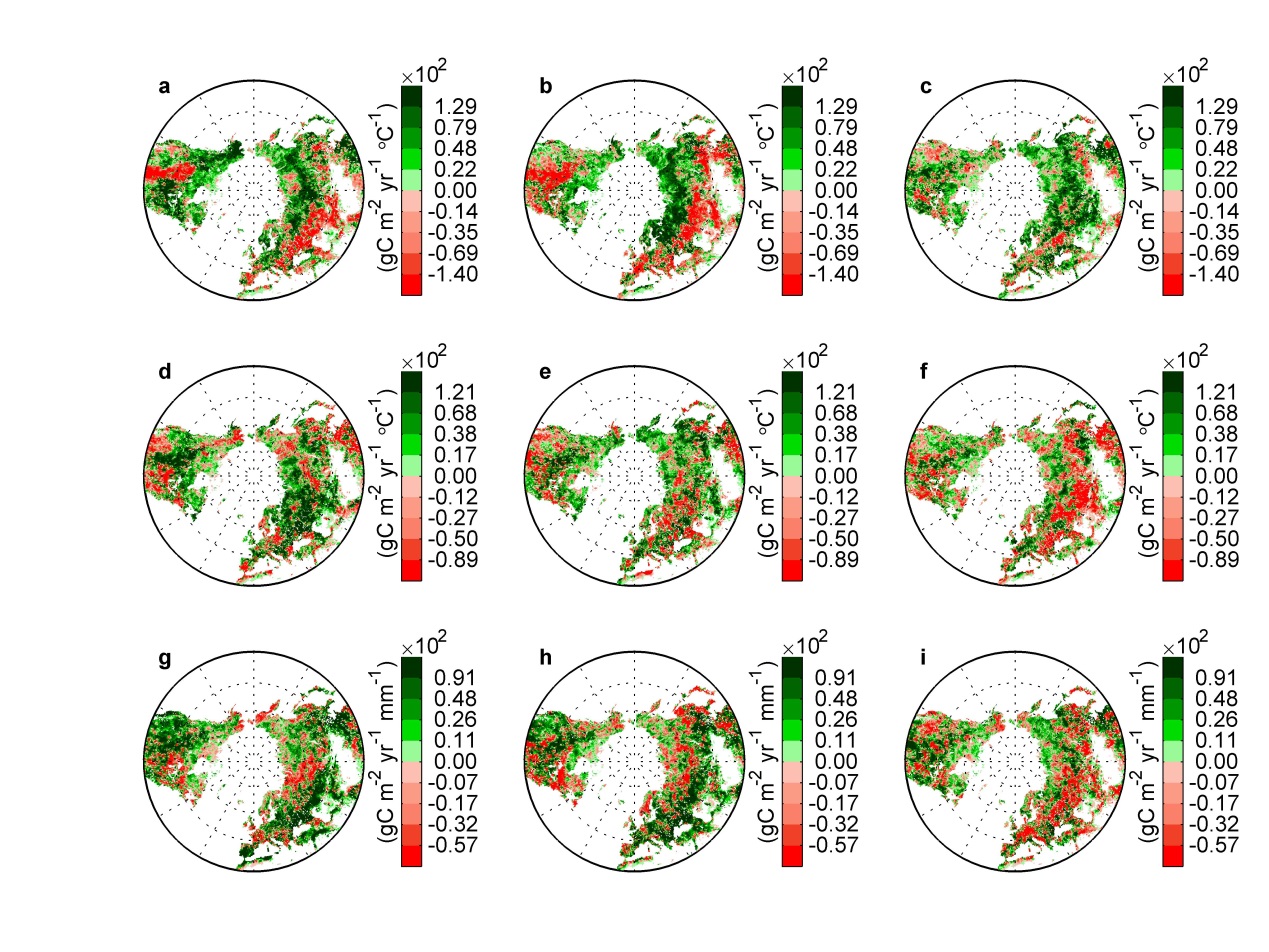


**Figure S8. Spatial patterns of the interannual sensitivity of growing-season (April-October) GPP to seasonal variations of maximum and minimum temperature and water availability index in mid- and high-latitudinal Northern Hemisphere during 1982-2008.** Interannual sensitivity of
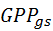
 (with unit of g C m^-2^ yr^-1^) to IAV of seasonal climate is estimated by ridge regression. Sensitivity of
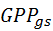
 to
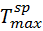
 (spring maximum temperature),
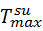
 (summer maximum temperature),
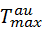
 (autumn maximum temperature),
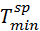
 (spring minimum temperature),
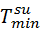
 (summer minimum temperature), and
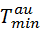
 (autumn minimum temperature) are shown in a-f, respectively. Sensitivity of
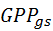
 to spring, summer and autumn *WAI* are shown in g-i, respectively. Blank regions are masked from our analysis (in detail see Methods). The map in this figure is created by MATLAB (R2012b).

**Figure S9**


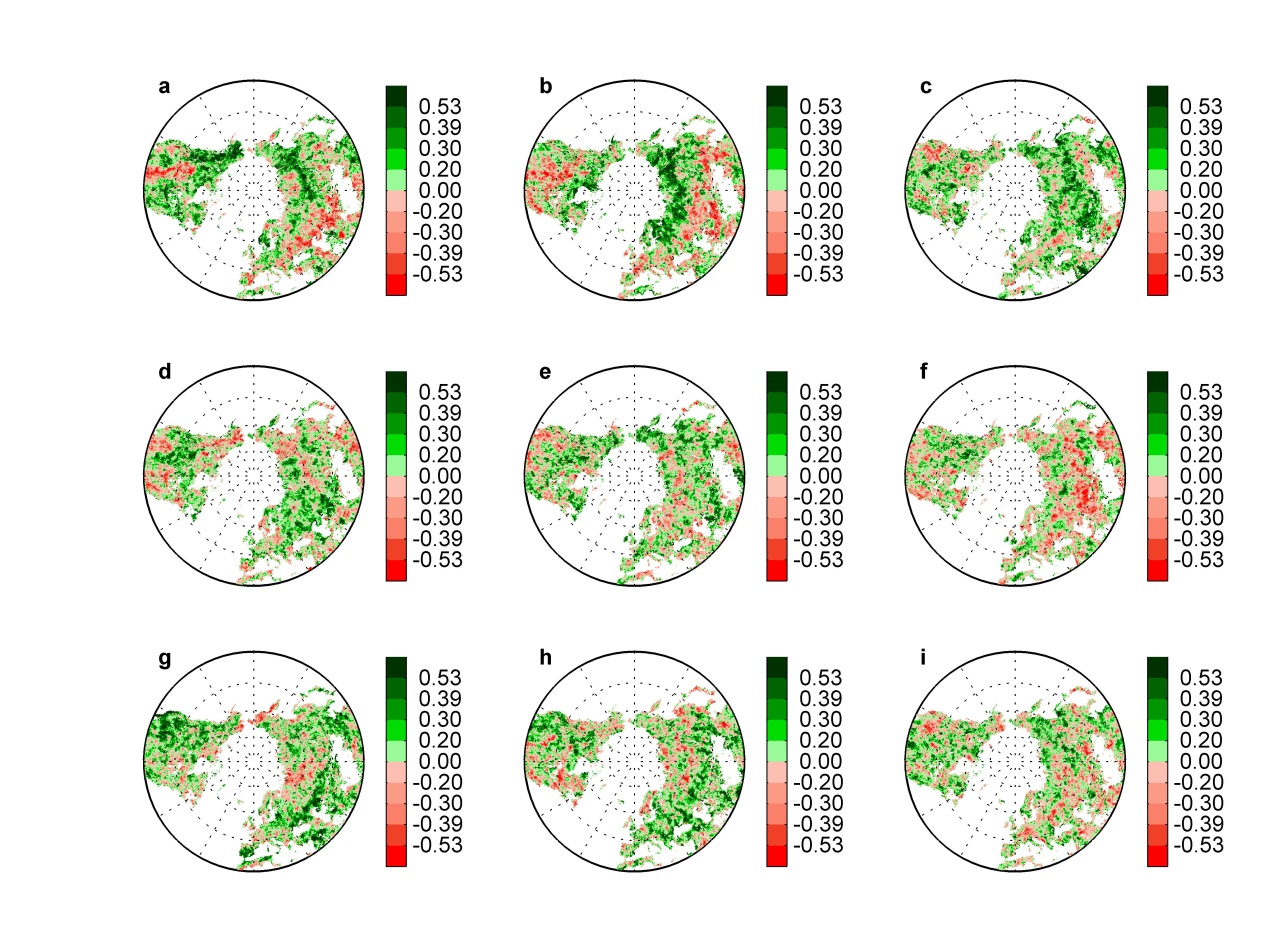


**Figure S9. Spatial patterns of the responses of growing-season (April-October) GPP to IAV of seasonal maximum and minimum temperature and water availability index in mid- and high-latitudinal Northern Hemisphere during 1982-2008.** Spearman partial correlation coefficients between growing-season GPP,
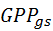
, and
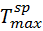
 (spring maximum temperature),
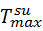
 (summer maximum temperature),
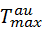
 (autumn maximum temperature),
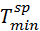
 (spring minimum temperature),
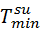
 (summer minimum temperature), and (autumn minimum temperature) are shown in a-f, respectively. Spearman partial correlation coefficients between and spring, summer and autumn *WAI* are shown in g-i, respectively. The Spearman correlation coefficients *R* ±0.53, ±0.39, ±0.30, and ±0.20 correspond to 1%, 5%, 10% and 20% significance level of student’s t-test, respectively. Blank regions are masked from our analysis (in detail see materials and methods). The map in this figure is created by MATLAB (R2012b).

**Figure S10**

**Figure S10. Latitudinal patterns of the responses of growing season (April-October) GPP to IAV of seasonal climate.** Latitudinal patterns of the median of partial correlation coefficients (*P_R_*) between and spring (green lines), summer (blue lines), and autumn (brown lines) maximum temperature (a), minimum temperature (b) and water availability index (c). Percentage of pixels (within every 0.5 degree interval) showing significant (*p*< 0.1) positive and negative correlation (*P_SR_*) of to spring (SP), summer (SU) and autumn (AU) maximum temperature (a), minimum temperature (b) and water availability index (c) are indicated by the filled green and red areas, respectively. The black lines in inlets of a, b and c show the sampling depths (N) within each 0.5 degree interval. The vertical red lines in a), b) and c) indicate the latitudes north than it the sample depths are lower than 50.

**Figure S11**

**Figure S11. Spatial patterns of the interannual responses of growing-season (May-October) NDVI to seasonal variations of maximum and minimum temperature and water availability index in mid- and high-latitudinal Northern Hemisphere during 1982-2008.** Spearman partial correlation coefficients between growing-season NDVI, , and (spring maximum temperature), (summer maximum temperature), (autumn maximum temperature), (spring minimum temperature), (summer minimum temperature), and (autumn minimum temperature) are shown in a-f, respectively. Spearman partial correlation coefficients between and spring, summer and autumn *WAI* are shown in g-i, respectively. The Spearman correlation coefficients *R* ±0.53, ±0.39, ±0.30, and ±0.20 correspond to 1%, 5%, 10% and 20% significance level of student’s t-test, respectively. Blank regions are masked from our analysis (in detail see materials and methods). The map in this figure is created by MATLAB (R2012b).

**Figure S12**

**Figure S12. Spatial patterns of the interannual responses of growing-season (April-September) NDVI to seasonal variations of maximum and minimum temperature and water availability index in mid- and high-latitudinal Northern Hemisphere during 1982-2008.** Spearman partial correlation coefficients between growing-season NDVI, , and (spring maximum temperature), (summer maximum temperature), (autumn maximum temperature), (spring minimum temperature), (summer minimum temperature), and (autumn minimum temperature) are shown in a-f, respectively. Spearman partial correlation coefficients between and spring, summer and autumn *WAI* are shown in g-i, respectively. The Spearman correlation coefficients *R* ±0.53, ±0.39, ±0.30, and ±0.20 correspond to 1%, 5%, 10% and 20% significance level of student’s t-test, respectively. Blank regions are masked from our analysis (in detail see materials and methods). The map in this figure is created by MATLAB (R2012b).

**Figure S13**

**Figure S13. Spatial patterns of the interannual responses of tree ring index to seasonal climate during 1960-2008 (if applicable) in mid- and high-latitudinal Northern Hemisphere.** Spearman partial correlation coefficients between tree ring index (TRI) and (spring maximum temperature), (summer maximum temperature), (autumn maximum temperature), (spring minimum temperature), (summer minimum temperature), and (autumn minimum temperature) are shown in a-f, respectively. Spearman partial correlation coefficients between TRI and spring, summer and autumn WAI are shown in g-i, respectively. The labels of 5%, 10% and 20% in colorbars indicate the corresponding significance level of student’s t-test. Detailed information about the TRI see Methods and Supplementary Table S3. The map in this figure is created by MATLAB (R2012b).

**Figure S14**

**Figure S14. Comparisons of mean water deficit in spring (green bars), summer (blue bars) and autumn (orange bars) for different climate zones (in detail see Methods).** The error bars indicate the standard deviations of regional water deficit. The smaller negative values indicate much more severe water deficit and vice versa.

**Figure S15**

**Figure S15. Interannual responses of growing-season (April-October) soil moisture to seasonal maximum and minimum temperature.** Spearman partial correlation coefficients between mean growing-season (April-October) remotely sensed soil moisture (SM) (in detail see Methods) and (spring maximum temperature), (summer maximum temperature), (autumn maximum temperature), (spring minimum temperature), (summer minimum temperature), and (autumn minimum temperature) are shown in a-f, respectively. The Spearman correlation coefficients *R* ±0.49, ±0.36, ±0.28, and ±0.19 correspond to 1%, 5%, 10% and 20% significance level of student’s t-test, respectively. Blank regions are masked from our analyses (in detail see Methods). The map in this figure is created by MATLAB (R2012b).

**Figure S16**

**Figure S16. Interannual response of total growing-season (April-October) water availability index to seasonal maximum and minimum temperature.** Spearman partial correlation coefficients between total growing-season (April-October) water availability index and (spring maximum temperature), (summer maximum temperature), (autumn maximum temperature), (spring minimum temperature), (summer minimum temperature), and (autumn minimum temperature) are shown in a-f, respectively. The Spearman correlation coefficients *R* ±0.49, ±0.36, ±0.28, and ±0.19 correspond to 1%, 5%, 10% and 20% significance level of student’s t-test, respectively. The map in this figure is created by MATLAB (R2012b).

**Figure 17**.

**Figure S17. Relationships between autumn-summer difference of water deficit and partial correlation coefficients between vegetation growth and seasonal** **.** Relationship between autumn-summer difference of water deficit (calculated as autumn water deficit minus summer water deficit, same for difference of partial correlation coefficients) and partial correlation coefficients between vegetation growth and seasonal for RegAR (a), RegTH (b) and RegTA (c) (in detail see Methods), respectively. Water deficit is calculated based on a simple water balance equation (in detail see Methods). Pixels with elevation lower than 75th percentile of elevation distribution in each of climate zones (in detail see Methods) are considered only to avoid the specific (e.g., mountain regions) characteristics in this relationship. The red and green lines in the figure are 75th and 50th percentile nonlinear (based on exponential equation) quantile regression lines, respectively. All nonlinear quantile fits are statistically significant (*p* < 0.05) except the 50th quantile regression for RegTH (*p* = 0.09) and RegTA (*p* = 0.12).

## References

1 Liang, E., Shao, X., Liu, H. & Eckstein, D. Tree-ring based PDSI reconstruction since AD 1842 in the Ortindag Sand Land, east Inner Mongolia*. Chinese Science Bullet*i**n** 52, 2715-2721 (2007).
